# The impact of timely community health services contact following hospital discharge on subsequent hospital use in England: retrospective observational study

**DOI:** 10.1186/s12916-026-05015-3

**Published:** 2026-07-15

**Authors:** Beth Parkinson, Matt Sutton, Lachlan Cameron, Rachel Meacock

**Affiliations:** Health Organisation, Policy and Economics (HOPE) Research Group, School of Health Sciences, The University of Manchester, Manchester, UK

**Keywords:** Community health care, Healthcare demand, Emergency care, Prevention, Avoidable admissions

## Abstract

**Background:**

Unplanned rehospitalisation is an undesirable outcome for patients and resource-constrained health systems. Community health services (CHS) support safe transitions home and recovery following hospital discharge, but evidence on their effectiveness in reducing rehospitalisation is limited. This study examined the impact of timely contact with CHS teams following hospital discharge on the odds of subsequent emergency hospital care.

**Methods:**

We used individual-level data from the Community Services Dataset on 63,019 individuals referred to 11 NHS Community Trust providers in England following hospital discharge between 1^st^ April and 31^st^ December 2019. Timeliness of CHS contact was defined as care being received within one day or between two and seven days post-discharge, compared to no contact within 30 days of discharge. Outcomes were emergency hospital readmission and emergency department (ED) attendance within 30 days of discharge. We used logistic regressions to estimate the association between timely CHS contact and hospital use, controlling for patient demographics, diagnosis and co-morbidities.

**Results:**

Individuals in our sample were 72.5 years of age on average with 2.3 comorbidities, and 52% were female. 52% received care within one day of discharge, 20% within two to seven days, and 28% had no contact within 30 days. Having a CHS contact within one day of hospital discharge was associated with 33% lower odds of ED attendance (OR: 0.674, 95% CI: 0.640 to 0.709) and 38% lower odds of readmission within 30 days of discharge (OR: 0.615, 95% CI: 0.583 to 0.649), compared to individuals with no contact in the follow up period. Receiving a CHS contact between two to seven days of discharge was associated with 20% lower odds of ED attendance (OR: 0.804, 95% CI: 0.756 to 0.855) and 22% lower odds of readmission (OR: 0.775, 95% CI: 0.728 to 0.825) within 30 days of discharge.

**Conclusions:**

Timely contact with CHS teams following discharge was associated with substantially lower odds of subsequent emergency hospital use. Whilst contact within two to seven days was beneficial for many patients, contact within one day of discharge was consistently associated with the largest reductions in hospital use across all patient groups.

## Background

Avoidable re-hospitalisation following discharge represents a persistent challenge for health systems worldwide. In the United States, approximately one in five Medicare patients are readmitted within 30 days of discharge [1], while in the English NHS this figure is around one in seven [2]. A systematic review estimated that a median of 27% of readmissions were potentially avoidable, meaning they were due to sub-optimal care either within the index admission or during follow-up [3]. These unplanned readmissions place significant strain on already stretched hospital resources and are distressing and disruptive events for patients. As a result, readmission rates are often used as an indicator of health care quality and efficiency [4, 5] as they can reflect inadequate inpatient care, discharge planning, or follow-up care. Reducing these events is therefore a key priority for many health systems [6], particularly given current concerns around capacity pressures and workforce shortages.

There is a large body of research investigating interventions to reduce hospital readmissions [7–9], including interventions during care transitions between hospital and home [10, 11], discharge planning [12], and post-discharge care at home [8, 13]. While receiving such interventions has generally been shown to lower readmission risk, evidence on the role of timing in post-discharge follow-up is more mixed [14, 15]. Many studies have shown that early physician follow-up is associated with a lower risk of readmission [16–23], but others report no association [24–26]. Differences in findings may reflect variation in the patient populations studied, with most investigating specific patient groups (often limited to those with specific chronic diseases, surgery admissions or elderly patients) and the definition of timely follow-up. Notably, most research has focused on the timing of follow-up with physicians in outpatient or primary care settings. There is a lack of evidence on the impact of timely follow-up from home-based, predominantly nurse-led, community health services such as community nursing and reablement teams.

Community health services (CHS) constitute a substantial component of NHS care in England, accounting for approximately 20% of the workforce [27], 10% of the NHS budget, and delivering over 100 million patient contacts each year [28]. CHS play a critical role in supporting patients to transition back home safely, providing rehabilitation, reablement, and intermediate care to help patients regain their independence following hospital discharge and prevent reattendance or readmission to hospital [27, 29, 30]. Patients referred to CHS are those deemed medically fit for discharge but are identified as having additional health care needs which require temporary support, assessment of long-term needs, or ongoing care at home, in line with NHS discharge pathways [31].

Despite their scale and importance to care delivery, relatively little is known about the provision of CHS or how they contribute to improved patient outcomes, including the prevention of hospital readmission. To date, studies have mostly relied on aggregate data to examine associations between CHS provision and hospital use at the system-level, showing that hospital activity is slightly lower in areas with higher levels of CHS activity [32], but finding no evidence of an association between the size of the community nursing workforce and hospital use in local areas in England [33]. A recent study exploiting variation in contact timing due to weekend delays found no evidence that short delays in CHS contact increased emergency department attendance among patients referred by primary care [34]. However, evidence on the link between community care receipt and hospital use at the individual-level remains limited, in particular regarding the importance of timely post-discharge follow-up care.

We use patient-level data to examine whether the timeliness of post-discharge contact from CHS affects the likelihood of subsequent emergency hospital attendance or admission. We evaluate this impact for the broad population of patients referred to a range of CHS by their acute provider following an initial hospital admission. Understanding whether and for whom timely follow-up contact impacts the risk of subsequent emergency hospital use is necessary to inform efforts to optimise post-discharge care and allocate community resources more effectively. This evidence is particularly timely as the recent NHS 10 Year Plan highlights a shift towards strengthening community-based care in England as one of its three major shifts [35], emphasising the need for a stronger evidence base on how these services can reduce hospital use.

## Methods

### Data

#### Data sources

We identified referrals to, and contacts with, community health services using the Community Services Dataset (CSDS). The CSDS is a patient-level administrative data set to which all providers of NHS-funded CHS in England are legally mandated to submit [36]. Providers are required to submit a patient list in each monthly reporting period, which includes all new referrals, open referrals and referrals which were closed during the reporting period. They are also required to report all care contacts that occurred during the reporting period.

Submission to the CSDS became legally mandated for all providers of adult community services in October 2017. As submission to the CSDS is a relatively new requirement, the completeness of this data has been improving over time. We therefore used the most recent extract available to us from the financial year 1^st^ April 2019 to 31^st^ March 2020.

We identified both the initial admission and any subsequent hospital use using Hospital Episode Statistics (HES) from the same financial year, 2019/20. HES is a patient-level administrative dataset recording detailed information on episodes of hospital-based care in England. For this study, we used data on admitted patient care and emergency department (ED) attendances. HES records patient demographic information (age, sex and ethnicity), clinical details (diagnoses, procedures and comorbidities), and information relating to the admission (dates of admission and discharge, and type of admission).

#### Sample construction

We focus on adult patients aged 18 and over referred to standalone CHS provider Trusts. These are large providers offering the full range of community services and are broadly comparable with each other, unlike the smaller, more specialised providers. We first restricted the data to referrals to the 18 standalone community Trusts in England. Patients can be referred to CHS from various sources, including a GP, self-referral or an acute hospital. To align with our focus on readmission avoidance, we restricted the sample to patients referred to CHS from an acute hospital Trust. Since this depended on the completeness of the referral source variable, we further restricted to the 11 Trusts for which fewer than 10% of records had missing referral source information.

We identified all new referrals opened between 1^st^ April and 31^st^ December 2019 with a referral source of ’06 - Acute Hospital Inpatient/Outpatient Department”. Restricting to referrals within this time window allowed us to follow up contacts and outcomes for 30-days whilst also ensuring that the follow-up period was not affected by the start of the COVID-19 pandemic.

Referrals from an acute Trust can be generated via either an inpatient or outpatient department. To identify referrals which were generated following an inpatient admission and therefore relevant to our analyses, we merged individuals’ CHS referral records with hospital admission records to identify a relevant index inpatient admission. We applied the following restrictions to identify a relevant index hospital admission for each CHS referral; the referral occurred after the date of hospital admission and the referral occurred within 30 days of discharge from hospital. Where a referral had multiple relevant inpatient admissions that met those criteria, the admission closest to the referral date was selected. This yielded a sample of 96,531 relevant CHS referrals with a unique matched admission episode. If individuals had multiple CHS referrals with matched admissions within the year, we then retained only the first referral per patient. These restrictions resulted in a sample of *n* = 80,138 referrals. Additional file 1: Figure S1 describes this sample selection process.

The follow-up period starts at date of discharge from the index admission, and we then follow all individuals for 30 days post-discharge.

#### Exposure: time to CHS contact

For each referral, we calculated the number of days from hospital discharge to the first CHS contact, excluding contacts marked as non-attended (e.g. cancellations, no shows, or late arrivals). We then created indicators for contact occurring within one day or between two and seven days of discharge, which form our exposures of interest. Individuals who were referred to CHS but had no CHS contact within the 30-day follow-up period were used as the comparison group.

Emergency hospital use soon after discharge does not preclude CHS contact in response to a referral but may alter its timing or likelihood. Individuals contacted between 8 and 30 days of discharge were therefore excluded from the comparison group (*n* = 17,119) due to concerns that any re-hospitalisation may influence CHS contact receipt during this later follow-up period. We conducted sensitivity analyses using all individuals who did not receive contact within 7 days as the control group to examine the sensitivity of our results to this exclusion and investigate the issue of early outcomes potentially influencing treatment exposure further in additional sensitivity analyses.

#### Outcomes: emergency hospital use within 30 days of discharge

The primary outcomes were emergency hospital use within 30 days of discharge from the index admission, specifically attendance at type 1 EDs (major consultant-led EDs, open 24-hours with full resuscitation facilities) and emergency readmissions (excluding planned, elective admission episodes). The 30-day follow-up period was chosen to align with national NHS quality indicators, capturing outcomes plausibly related to the index admission and reflecting the success of care in supporting patient recovery [37]. We also examined a subset of potentially preventable emergency readmissions for ambulatory care sensitive conditions (ACSCs). These are conditions for which hospitalisation is considered potentially preventable with appropriate primary and community care [38], using definitions from NHS performance indicators [39].

#### Control variables

We controlled for a detailed set of individual characteristics using information obtained from the patient’s index admission record. This included interactions of sex and age group (ten-year age bands) and ethnic group (five categories). Primary diagnosis was classified using the 19 higher-level diagnosis clusters from the ICD-10 to SHMI diagnostic group lookup [40]. To account for patient complexity, we included indicators for the presence of each of the 30 Elixhauser comorbidities on the patient’s index admission record [41]. We also controlled for socio-economic deprivation using the Index of Multiple Deprivation (IMD) decile of the Lower layer Super Output Area (LSOA) of the patient’s residence [42]. This index incorporates multiple indicators across seven domains of deprivation: income, employment, education, health, crime, barriers to housing and services, and living environment (e.g. air quality).

We adjusted for whether the index admission was elective or emergency, as this may influence both the timeliness of CHS contact and post-discharge outcomes. We controlled for an indicator of whether the patient was discharged from their index admission to their usual place of residence. Additional controls included the calendar month and day of the week of discharge from hospital, to account for any seasonal and weekday-related variation in care provision or delivery. We also included CHS and acute provider fixed effects to account for differences in average admission rates that may be caused by differences in organisational culture and service delivery across providers. .

### Statistical methods

We first examined descriptive statistics of patient characteristics, severity indicators, and outcomes stratified by CHS contact timing category. We then used separate logistic regression models to estimate the association between timely CHS contact and the odds of each type of hospital use outcome within 30 days post-discharge: ED attendance, emergency readmission, and ACSC readmission. We estimated a series of these regression models, presenting the results as groups of covariates are sequentially added to show the impact of covariate adjustment on the estimated associations. Model 1 contained only the CHS contact exposure group indicators of interest, then models 2-5 sequentially added covariates capturing demographic characteristics, diagnoses, month and day of discharge, and provider indicators, with model 5 representing the fully adjusted specification. We also presented these results graphically using coefficient plots showing the estimated associations and 95% confidence intervals from models 1-5 for each outcome.

#### Supplementary analyses

We conducted several supplementary analyses to examine whether significant relationships between timely CHS contact and hospital use are present within different patient sub-groups, stratifying the analyses by: age group ( < 65 and 65 + years); index admission type (emergency vs elective); and the eight most common primary diagnoses.

We examined the robustness of our results to changes in the follow-up window over which hospital use outcomes are assessed. Emergency hospital use soon after discharge does not preclude CHS contact in response to the referral, but may alter its timing or likelihood, potentially introducing dynamics similar to immortal-time bias [43]. This is less of an issue for ED attendances than for readmissions, which may be more likely to disrupt or delay CHS contact since patients may be temporarily unable to receive CHS care during an inpatient stay. We therefore examined the relationship between timely CHS contact in the first week following discharge and hospital use in the subsequent non-overlapping period, 8 to 30 days post-discharge. We first undertook this analysis for all patients in our sample, then conducted a landmark analysis excluding individuals who experience the hospital outcome of interest on or before the fixed landmark date of 7 days post-discharge [44]. We also conducted analysis examining hospital use outcomes within 90-days of-discharge, for the sub-sample of patients referred to CHS in the first seven months of our analytical period (between April and October 2019).

We also ran separate sensitivity analyses using individuals not contacted within 7 days of discharge as the comparator group to examine the robustness of our primary analysis to alternative definitions of the comparator. Finally, we performed additional sensitivity analysis excluding those discharged before 1^st^ May 2019, to allow a minimum clean run-in period of 30 days for all patients in our sample.

## Results

### Patient characteristics and outcomes by timing of community health service contact

Amongst the sample of patients referred to CHS by their acute hospital provider following a hospital admission, 41% received a care contact within one day of discharge from hospital. A further 15.7% received a community care contact within two to seven days of discharge, 21.4% between 8 and 30 days, whilst 21.9% of the sample did not receive a care contact within the follow up period (Additional file 1: Table S1). Individuals who received CHS care contact within one day of discharge were on average older (75 years) and had greater clinical complexity as indicated by a higher mean number of Elixhauser comorbidities (2.5) compared with those with a first care contact between two and seven days (70.0 years, 2.2 comorbidities) and those with no care contact within the follow up period (69.6 years, 2.1 comorbidities) (Table 1). The proportion of index admissions which occurred in an emergency rather than electively was higher amongst the group of patients who received a CHS care contact within one day of discharge (80.7%), compared with those who first received a CHS contact between two and seven days (65.0%) and those who did not receive a CHS contact within 30 days of discharge (65.6%). Unadjusted rates of subsequent hospital use varied across exposure groups of time to first CHS care contact (Table 1). ED attendance occurred in 19.2% of patients seen within one day of discharge, 18.8% of those seen first by CHS between two and seven days following discharge, and 23.8% of patients who had no CHS care contact within the follow up period. The corresponding 30-day emergency readmission rates were 17.8%, 18.1%, and 22.7% respectively, while ACSC admission rates were 4.9%, 4.7% and 5.8% respectively.

**Table 1:** Descriptive statistics

	Contact within 1 day (*N* = 32,866)	Contact between 2 and 7 days (*N* = 12,579)	No contact within 30 days (*N* = 17,574)	Total (*N* = 63,019)
ED attendance within 30 days of discharge	6298 (19.2%)	2364 (18.8%)	4191 (23.8%)	12853 (20.4%)
Emergency re-admission within 30 days of discharge	5853 (17.8%)	2272 (18.1%)	3984 (22.7%)	12109 (19.2%)
Re-admission for ACSC within 30 days of discharge	1597 (4.9%)	591 (4.7%)	1014 (5.8%)	3202 (5.1%)
Age (Mean (SD))	75.0 (15.1)	70.0 (16.7)	69.6 (17.9)	72.5 (16.4)
Age group				
Aged 18-30	519 (1.6%)	409 (3.3%)	794 (4.5%)	1722 (2.7%)
Aged 30-39	658 (2.0%)	452 (3.6%)	813 (4.6%)	1923 (3.1%)
Aged 40-49	1162 (3.5%)	653 (5.2%)	957 (5.4%)	2772 (4.4%)
Aged 50-59	2604 (7.9%)	1418 (11.3%)	1788 (10.2%)	5810 (9.2%)
Aged 60-69	4478 (13.6%)	2287 (18.2%)	2802 (15.9%)	9567 (15.2%)
Aged 70-79	8567 (26.1%)	3388 (26.9%)	4554 (25.9%)	16509 (26.2%)
Aged 80-89	10730 (32.6%)	3036 (24.1%)	4412 (25.1%)	18178 (28.8%)
Aged 90-99	4067 (12.4%)	910 (7.2%)	1431 (8.1%)	6408 (10.2%)
Aged 100+	81 (0.2%)	26 (0.2%)	23 (0.1%)	130 (0.2%)
Sex				
Male	15282 (46.5%)	6494 (51.6%)	8489 (48.3%)	30265 (48.0%)
Female	17584 (53.5%)	6085 (48.4%)	9085 (51.7%)	32754 (52.0%)
Major ethnic group				
Unknown	671 (2.0%)	421 (3.3%)	499 (2.8%)	1591 (2.5%)
White	28800 (87.6%)	10691 (85.0%)	15004 (85.4%)	54495 (86.5%)
Mixed	473 (1.4%)	197 (1.6%)	415 (2.4%)	1085 (1.7%)
Asian	295 (0.9%)	128 (1.0%)	261 (1.5%)	684 (1.1%)
Black	2627 (8.0%)	1142 (9.1%)	1395 (7.9%)	5164 (8.2%)
Primary diagnosis of index admission				
Blood disorders	222 (0.7%)	79 (0.6%)	204 (1.2%)	505 (0.8%)
Cancer	2707 (8.2%)	1121 (8.9%)	1858 (10.6%)	5686 (9.0%)
Digestive system	1892 (5.8%)	564 (4.5%)	994 (5.7%)	3450 (5.5%)
Endocrine, nutritional and metabolic disease	1052 (3.2%)	282 (2.2%)	477 (2.7%)	1811 (2.9%)
Heart	2528 (7.7%)	2046 (16.3%)	1956 (11.1%)	6530 (10.4%)
Infectious disease	1082 (3.3%)	256 (2.0%)	408 (2.3%)	1746 (2.8%)
Injuries / fractures	5175 (15.7%)	1726 (13.7%)	2371 (13.5%)	9272 (14.7%)
Liver	436 (1.3%)	139 (1.1%)	216 (1.2%)	791 (1.3%)
Mental illness	659 (2.0%)	96 (0.8%)	262 (1.5%)	1017 (1.6%)
Musculoskeletal disorders	3454 (10.5%)	2071 (16.5%)	2095 (11.9%)	7620 (12.1%)
Nervous system	767 (2.3%)	235 (1.9%)	447 (2.5%)	1449 (2.3%)
Other	1718 (5.2%)	502 (4.0%)	780 (4.4%)	3000 (4.8%)
Perinatal	24 (0.1%)	17 (0.1%)	16 (0.1%)	57 (0.1%)
Pregnancy related	118 (0.4%)	116 (0.9%)	536 (3.0%)	770 (1.2%)
Renal / genitourinary	2555 (7.8%)	781 (6.2%)	1285 (7.3%)	4621 (7.3%)
Respiratory	3837 (11.7%)	1074 (8.5%)	1736 (9.9%)	6647 (10.5%)
Sense organs	335 (1.0%)	147 (1.2%)	267 (1.5%)	749 (1.2%)
Skin	1295 (3.9%)	333 (2.6%)	430 (2.4%)	2058 (3.3%)
Vascular / circulatory	3010 (9.2%)	994 (7.9%)	1236 (7.0%)	5240 (8.3%)
Count of Elixhauser comorbidities (Mean (SD))	2.5 (1.7)	2.2 (1.7)	2.1 (1.7)	2.3 (1.7)
Presence of Elixhauser comorbidities				
Congestive heart failure	4064 (12.4%)	1271 (10.1%)	1648 (9.4%)	6983 (11.1%)
Cardiac arrhythmia	8708 (26.5%)	2728 (21.7%)	3766 (21.4%)	15202 (24.1%)
Valvular disease	2625 (8.0%)	1055 (8.4%)	1132 (6.4%)	4812 (7.6%)
Pulmonary circulation disorders	488 (1.5%)	164 (1.3%)	182 (1.0%)	834 (1.3%)
Peripheral vascular disease	1665 (5.1%)	552 (4.4%)	694 (3.9%)	2911 (4.6%)
Hypertension uncomplicated	16311 (49.6%)	5824 (46.3%)	7884 (44.9%)	30019 (47.6%)
Hypertension complicated	44 (0.1%)	17 (0.1%)	30 (0.2%)	91 (0.1%)
Paralysis	1085 (3.3%)	293 (2.3%)	441 (2.5%)	1819 (2.9%)
Other neurologic disorders	2817 (8.6%)	812 (6.5%)	1269 (7.2%)	4898 (7.8%)
Chronic pulmonary disease	7095 (21.6%)	2479 (19.7%)	3451 (19.6%)	13025 (20.7%)
Diabetes uncomplicated	6420 (19.5%)	2271 (18.1%)	3114 (17.7%)	11805 (18.7%)
Diabetes complicated	1062 (3.2%)	294 (2.3%)	523 (3.0%)	1879 (3.0%)
Hypothyroidism	3055 (9.3%)	972 (7.7%)	1356 (7.7%)	5383 (8.5%)
Renal failure	5100 (15.5%)	1558 (12.4%)	2252 (12.8%)	8910 (14.1%)
Liver disease	890 (2.7%)	282 (2.2%)	478 (2.7%)	1650 (2.6%)
Peptic ulcer disease excluding bleeding	144 (0.4%)	45 (0.4%)	45 (0.3%)	234 (0.4%)
Lymphoma	203 (0.6%)	69 (0.5%)	104 (0.6%)	376 (0.6%)
Metastatic cancer	1521 (4.6%)	491 (3.9%)	900 (5.1%)	2912 (4.6%)
Solid tumor without metastasis	1875 (5.7%)	573 (4.6%)	845 (4.8%)	3293 (5.2%)
Rheumatoid arthritis/collagen	1817 (5.5%)	657 (5.2%)	882 (5.0%)	3356 (5.3%)
Coagulopathy	357 (1.1%)	89 (0.7%)	160 (0.9%)	606 (1.0%)
Obesity	2719 (8.3%)	1365 (10.9%)	1605 (9.1%)	5689 (9.0%)
Weight loss	574 (1.7%)	132 (1.0%)	255 (1.5%)	961 (1.5%)
Fluid and electrolyte disorders	4521 (13.8%)	1059 (8.4%)	1679 (9.6%)	7259 (11.5%)
Blood loss anemia	35 (0.1%)	10 (0.1%)	14 (0.1%)	59 (0.1%)
Deficiency anemia	1457 (4.4%)	387 (3.1%)	541 (3.1%)	2385 (3.8%)
Alcohol abuse	1331 (4.0%)	428 (3.4%)	609 (3.5%)	2368 (3.8%)
Drug abuse	169 (0.5%)	65 (0.5%)	121 (0.7%)	355 (0.6%)
Psychoses	273 (0.8%)	75 (0.6%)	135 (0.8%)	483 (0.8%)
Depression	3034 (9.2%)	1070 (8.5%)	1528 (8.7%)	5632 (8.9%)
Index of Multiple Deprivation (IMD) Decile				
1 (most deprived)	1751 (5.3%)	664 (5.3%)	907 (5.2%)	3322 (5.3%)
2	2532 (7.7%)	939 (7.5%)	1333 (7.6%)	4804 (7.6%)
3	3001 (9.1%)	1099 (8.7%)	1772 (10.1%)	5872 (9.3%)
4	3358 (10.2%)	1340 (10.7%)	1955 (11.1%)	6653 (10.6%)
5	3927 (11.9%)	1528 (12.1%)	2397 (13.6%)	7852 (12.5%)
6	4274 (13.0%)	1577 (12.5%)	2289 (13.0%)	8140 (12.9%)
7	3370 (10.3%)	1331 (10.6%)	1957 (11.1%)	6658 (10.6%)
8	3534 (10.8%)	1379 (11.0%)	1786 (10.2%)	6699 (10.6%)
9	3613 (11.0%)	1368 (10.9%)	1821 (10.4%)	6802 (10.8%)
10 (least deprived)	3506 (10.7%)	1354 (10.8%)	1357 (7.7%)	6217 (9.9%)
Index admission mode was emergency	26519 (80.7%)	8176 (65.0%)	11520 (65.6%)	46215 (73.3%)
Discharged to usual place of residence	28973 (88.2%)	11771 (93.6%)	15664 (89.1%)	56408 (89.5%)
Month of discharge				
Apr-19	4635 (14.1%)	1590 (12.6%)	1703 (9.7%)	7928 (12.6%)
May-19	4720 (14.4%)	1584 (12.6%)	1781 (10.1%)	8085 (12.8%)
Jun-19	4109 (12.5%)	1372 (10.9%)	1637 (9.3%)	7118 (11.3%)
Jul-19	3930 (12.0%)	1428 (11.4%)	2226 (12.7%)	7584 (12.0%)
Aug-19	3512 (10.7%)	1384 (11.0%)	2042 (11.6%)	6938 (11.0%)
Sep-19	2963 (9.0%)	1384 (11.0%)	2020 (11.5%)	6367 (10.1%)
Oct-19	3222 (9.8%)	1341 (10.7%)	2153 (12.3%)	6716 (10.7%)
Nov-19	3031 (9.2%)	1356 (10.8%)	2333 (13.3%)	6720 (10.7%)
Dec-19	2744 (8.3%)	1140 (9.1%)	1679 (9.6%)	5563 (8.8%)
Day of week of discharge				
Sunday	1646 (5.0%)	660 (5.2%)	940 (5.3%)	3246 (5.2%)
Monday	4844 (14.7%)	1626 (12.9%)	2655 (15.1%)	9125 (14.5%)
Tuesday	6127 (18.6%)	1957 (15.6%)	3151 (17.9%)	11235 (17.8%)
Wednesday	6238 (19.0%)	1942 (15.4%)	3037 (17.3%)	11217 (17.8%)
Thursday	6164 (18.8%)	1829 (14.5%)	3024 (17.2%)	11017 (17.5%)
Friday	5603 (17.0%)	2958 (23.5%)	3225 (18.4%)	11786 (18.7%)
Saturday	2244 (6.8%)	1607 (12.8%)	1542 (8.8%)	5393 (8.6%)

### Timeliness of CHS contact and emergency hospital use

**Fig. 1:**
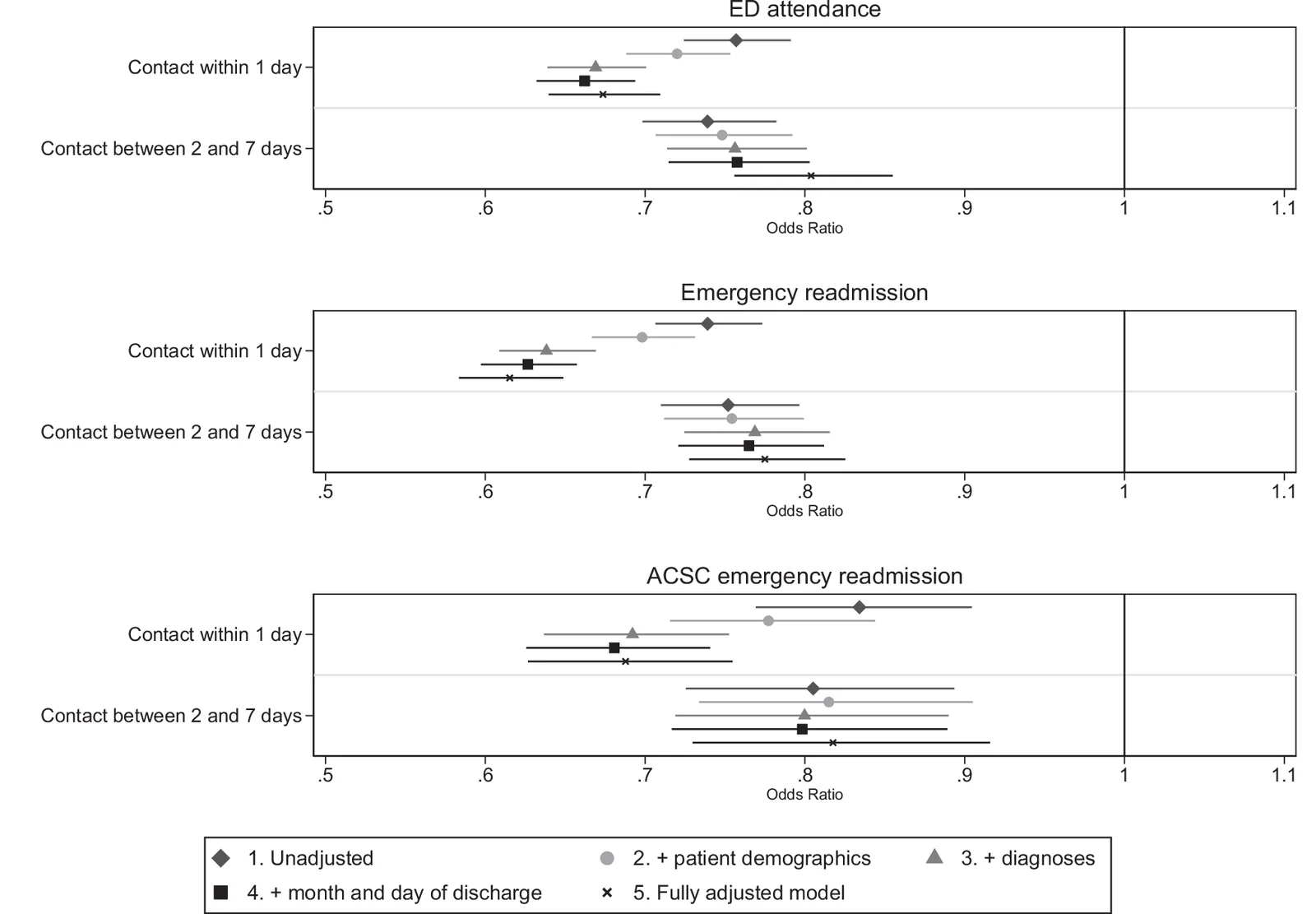
Coefficient plots of time to community health services contact on hospital use

Table 2 and Fig. 1 present the logistic regression estimates of the associations between CHS contact timing and subsequent hospital use across models with increasing amounts of covariate adjustment. In all specifications, care contact within one day or within two and seven days of discharge was associated with significantly lower odds of ED attendance, emergency readmission, and ACSC readmission compared with individuals who received no contact within the follow up period (Fig. 1). The associations were larger for patients who received a care contact within one day of discharge compared to those contacted within two and seven days (Fig. 1).

**Table 2:** Logistic regressions of time to community health services contact on hospital use

Outcome		1 (Unadjusted)	2	3	4	5 (Fully adjusted)
		OR [95% CI]	OR [95% CI]	OR [95% CI]	OR [95% CI]	OR [95% CI]
Emergency department attendance within 30 days	Ref: No contact within follow-up period	-	-	-	-	-
	Contact within 1 day of discharge	0.757^***^	0.720^***^	0.669^***^	0.662^***^	0.674^***^
		[0.724,0.791]	[0.688,0.753]	[0.639,0.701]	[0.632,0.694]	[0.640,0.709]
	Contact between 2 and 7 days	0.739^***^	0.748^***^	0.756^***^	0.758^***^	0.804^***^
		[0.698,0.782]	[0.707,0.792]	[0.714,0.801]	[0.715,0.803]	[0.756,0.855]
	C-statistic	0.531	0.575	0.621	0.624	0.637
Emergency readmission within 30 days	Ref: No contact within follow-up period	-	-	-	-	-
	Contact within 1 day of discharge	0.739^***^	0.698^***^	0.638^***^	0.627^***^	0.615^***^
		[0.706,0.773]	[0.667,0.731]	[0.609,0.669]	[0.597,0.657]	[0.583,0.649]
	Contact between 2 and 7 days	0.752^***^	0.754^***^	0.769^***^	0.765^***^	0.775^***^
		[0.710,0.796]	[0.712,0.799]	[0.725,0.815]	[0.721,0.812]	[0.728,0.825]
	C-statistic	0.532	0.577	0.645	0.648	0.654
Preventable admission within 30 days	Ref: No contact within follow-up period	-	-	-	-	-
	Contact within 1 day of discharge	0.834^***^	0.777^***^	0.692^***^	0.681^***^	0.688^***^
		[0.769,0.904]	[0.716,0.844]	[0.637,0.752]	[0.626,0.741]	[0.627,0.755]
	Contact between 2 and 7 days	0.805^***^	0.815^***^	0.800^***^	0.798^***^	0.818^***^
		[0.726,0.893]	[0.734,0.905]	[0.719,0.890]	[0.717,0.889]	[0.730,0.916]
	C-statistic	0.522	0.592	0.705	0.708	0.716
	Other covariates included in regression models:					
	Patient demographics	No	Yes	Yes	Yes	Yes
	Diagnoses	No	No	Yes	Yes	Yes
	Month and day of discharge	No	No	No	Yes	Yes
	Provider indicators	No	No	No	No	Yes

*N* = 63,019. Individuals with no contact during the follow-up period are the comparator group. Models 1-5 show the impact of covariate adjustment on the estimated associations, when the groups of covariates listed in the bottom panels of the table are sequentially added. Patient demographics covariates: age group (ten-year age bands), sex, ethnic group and IMD decile. Diagnosis covariates: 19 primary diagnosis clusters, 30 indicators for the presence of each Elixhauser comorbidity, indicator for index admission being an emergency, whether discharged to usual place of residence. Month and day discharge covariates: indicators for calendar month and day of week of discharge. Provider indicators: indicators for CHS provider of referral and acute care provider of index admission. Coefficients are odds ratios. 95% confidence intervals in brackets. C-statistic (area under the receiver operating characteristic curve) measures the model’s discriminatory power

*ED* emergency department, *ACSC* ambulatory care sensitive conditions

^*^p < 0.05, ^**^p < 0.01, ^***^p < 0.001

For ED attendance, the unadjusted odds ratio for one-day contact was 0.757 (95% CI: 0.724 to 0.791), indicating 24% lower odds compared to individuals with no CHS contact within the follow up period (Table 2, panel 1, column 1). This estimate increased in magnitude when adjusting for patient characteristics (column 2), patient diagnoses and comorbidities (column 3) and the timing of the discharge (column 4). In the fully adjusted model (column 5), the odds ratio was 0.674 (95% CI: 0.640 to 0.709), indicating a 33% lower odds of ED attendance associated with CHS contact within one day of discharge. Contact between two and seven days of discharge was associated with a similar magnitude effect on ED use in the unadjusted models, with an odds ratio of 0.739 (95% CI: 0.698 to 0.782), or 26% lower odds. However, the magnitude of the estimated association reduced with the addition of covariates (columns 2-5). Contact between two and seven days was associated with 20% lower odds of ED use, compared with individuals who did not receive a contact (column 5, OR: 0.804, 95% CI: 0.756 to 0.855) in the fully adjusted model.

A similar pattern was observed for emergency readmissions (Table 2, panel 2) and emergency readmissions for ACSCs (Table 2, panel 3). In the fully adjusted models, one-day contact was associated with 38% lower odds of emergency readmission (OR: 0.615, 95% CI: 0.583 to 0.649), and two-to-seven day contact was associated with 22% lower odds of readmission (OR: 0.775, 95% CI: 0.728 to 0.825), compared with no contact. Similarly, individuals who received a contact within one day of discharge had a 31% lower odds of having an emergency readmission for an ACSC (OR: 0.688, 95% CI: 0.627 to 0.755), whilst those contacted between two and seven days experienced a 18% lower odds of an emergency readmission for an ACSC (OR: 0.818, 95% CI: 0.730 to 0.916). Regression coefficients for all other covariates included in the models are presented in Additional file 1: Table S2.

### Subgroup and robustness analyses

**Fig. 2:**
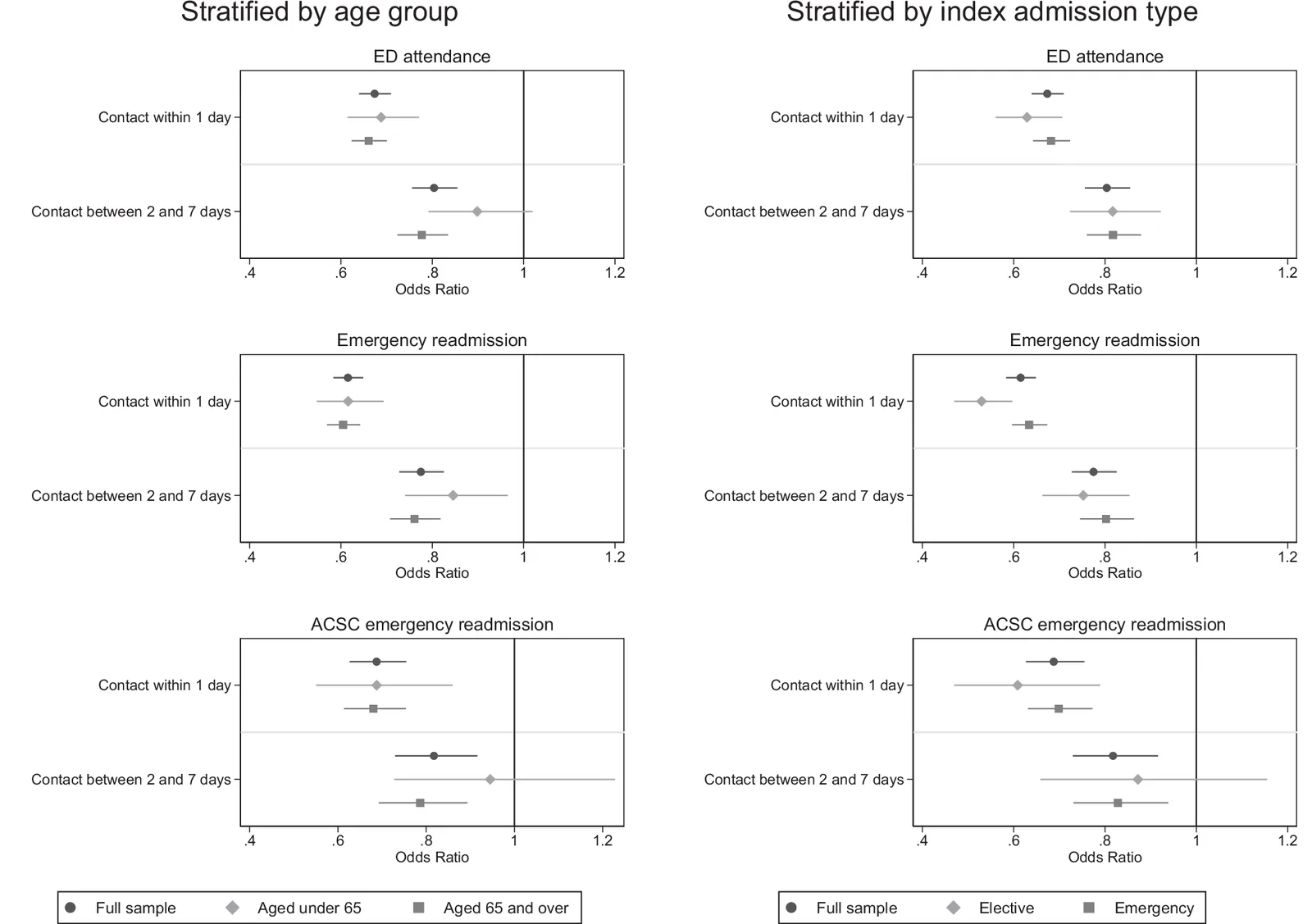
Coefficient plot of CHS timing and hospital use stratified by age group and index admission type

Figure 2 and Additional file 1: Table S3 present the logistic regression estimates stratified by age and index admission type. Care contact within one day of discharge was associated with significantly reduced odds of hospital use in both age groups, with similar magnitudes after covariate adjustment. Contact within two and seven days was also associated with reduced hospital use in both groups, but estimates were generally more precise among patients aged 65 years and over than among the smaller group of those aged under 65.

When stratified by index admission type, timely CHS contact was associated with significantly lower odds of hospital use for both elective and emergency index admissions, with estimates appearing larger amongst individuals whose initial admission was elective.

**Fig. 3:**
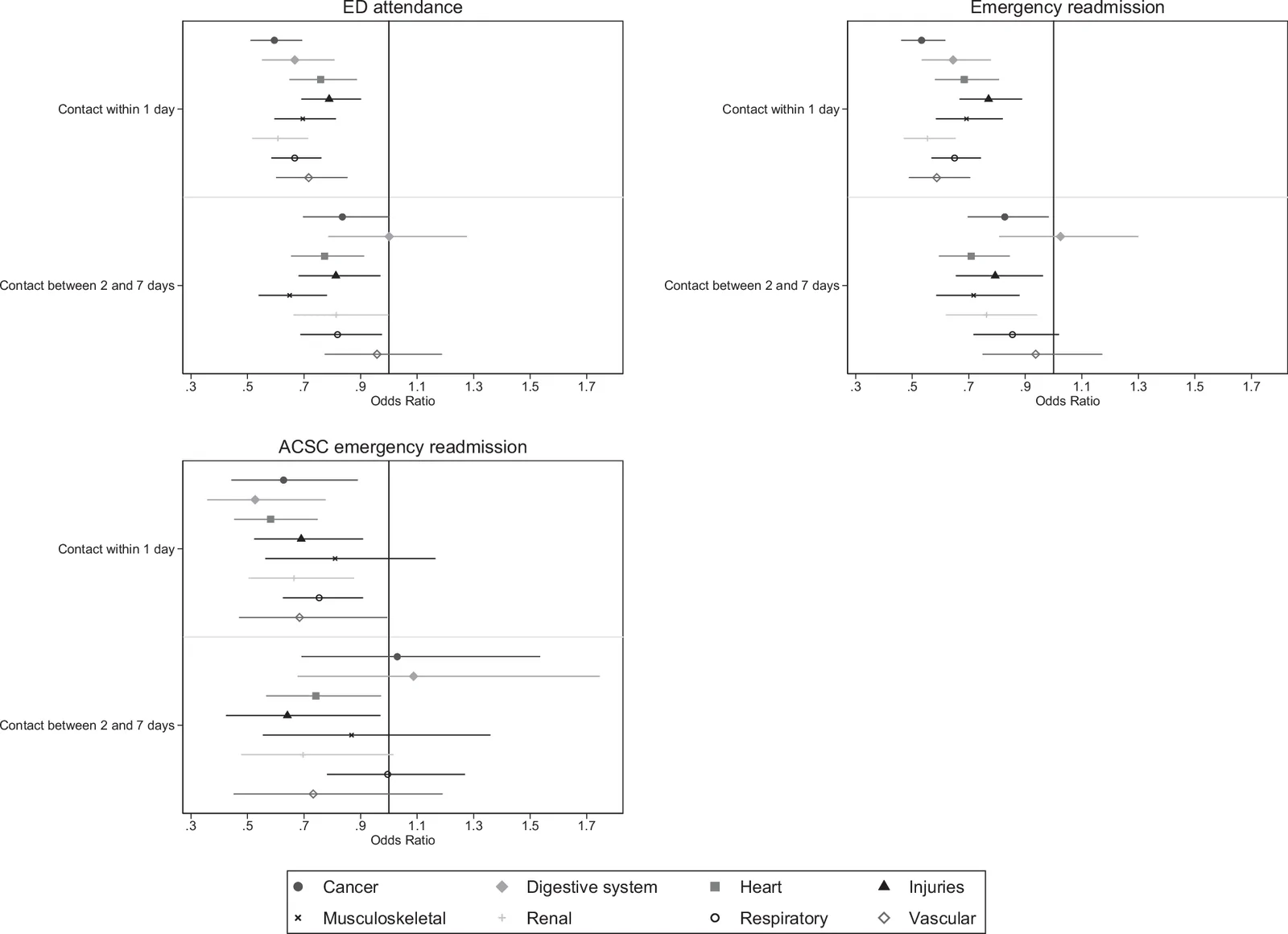
Coefficient plot of community health services timing and hospital use stratified by primary diagnosis

Figure 3 and Additional file 1: Table S4 show the regressions stratified by the eight most common primary diagnoses of index admission. Contact within one day of discharge was associated with significantly lower odds of ED use and emergency readmission across all eight diagnoses, with the largest estimates observed in odds of ED use for cancer (OR:0.595) or renal diagnoses (OR:0.608), and in the odds of readmission for cancer (OR:0.533), renal (OR: 0.554) or vascular diagnoses (OR: 0.587). Contact within one day of discharge was also associated with significantly lower odds of readmission for an ACSC across seven of the eight primary diagnoses when examined individually. Associations for contact between two and seven days and hospital use were more variable across primary diagnosis groups.

Additional file 1: Table S5 presents the results examining hospital use outcomes in the period 8 to 30 days following discharge. Consistent with our main analysis, contact within one day or within two and seven days of discharge was associated with significantly lower odds of ED attendance, emergency readmission, and ACSC readmission compared with individuals who received no contact within 30 days of discharge. In adjusted models, odds ratios ranged from 0.655 to 0.701 across outcomes for contact within one day, and from 0.604 to 0.688 for contact between two and seven days. Findings were similar in the landmark analyses excluding patients who experienced the hospital outcome on or before day 7, where odds ratios ranged from 0.620 to 0.678 and 0.606 and 0.687 across outcomes, respectively.

Significant reduction in the odds of all hospital use outcomes were still observed at 90 days post-discharge for those receiving a CHS contact within one day (ED attendance OR: 0.751, emergency readmission OR: 0.717, ACSC readmission OR: 0.788) and within two to seven days of discharge (ED attendance OR: 0.820, emergency readmission OR: 0.817, ACSC readmission OR: 0.836), amongst the subsample of patients whom we were able to follow-up for this extended period (Additional file 1: Table S6).

Additional file 1: Table S7 presents the results when including the group of individuals contacted between eight and thirty days post-discharge in the comparison group. The estimated coefficients associated with one-day and two to seven-day contact are larger than in our main analyses, as the previously excluded group has much higher hospital use. Finally, the results were also robust to excluding those discharged before 1^st^ May 2019, to allow a minimum discharge-referral episode free run-in period of 30 days (Additional file 1: Table S8).

## Discussion

### Summary of findings

This study investigated the impact of timely post-discharge follow-up with CHS on subsequent hospital use within 30 days of discharge. Our findings suggest that the timeliness of delivering post-discharge CHS care is associated with a markedly lower odds of ED attendance, emergency readmission, and readmission for ambulatory care-sensitive conditions, even after adjusting for patient complexity.

The majority of individuals who were referred received contact with CHS within seven days of discharge from hospital; 41% within one day of discharge and a further 15.7% between two and seven days. Patients who received a CHS contact within one day of discharge had 33% lower odds of ED use and 38% lower odds of readmission within 30 days, compared to those who were referred to community services following hospitalisation but received no CHS contact within the follow-up period. Patient complexity was higher amongst those receiving timely care contacts, suggesting services prioritise higher-risk patients for earlier care contact. Despite greater complexity, these patients had lower subsequent hospital use than those who did not receive a care contact during the follow-up period. Contact within two to seven days was also associated with significant reductions in subsequent hospital use. Whilst still substantial in magnitude at around 20% lower odds than no contact, these effects were smaller than for contact within one day, and not statistically significant in some subgroups (younger patient and certain diagnoses). Significant reductions in the odds of hospital use were also observed 90 days post-discharge.

Subgroup analyses showed broadly consistent associations across age groups, admission types, and diagnoses, with particularly large effects for patients with cancer, renal, or vascular conditions. Our analysis focused on the eight most common diagnoses, which may not necessarily represent those with the greatest capacity to benefit from timely care, but it provides insight into the relationship between timely CHS follow-up and hospital use for the conditions most frequently seen in practice. Overall, timely CHS contact, particularly within the first day after discharge, appears to play an important role in reducing avoidable hospital use.

### Comparison with previous literature

These findings are consistent with prior research demonstrating the benefits of early post-discharge follow-up on reducing hospital use. Several studies have shown that physician follow-up, in outpatient or primary care settings, was associated with a lower risk of readmission [18–23, 26, 45]. A systematic review and meta-analysis found that outpatient follow-up visits were associated with a 21% lower risk of readmission for heart failure, COPD, myocardial infarction, and stroke patients [23]. However, studies vary in the definition of timely follow-up, and few have explored the optimal timing of follow-up care in terms of readmission prevention. One study compared the impact of outpatient physician follow-up at various time points after discharge on readmission within 30 days, finding that follow-up within 7 days was most beneficial for high-risk patients, with a 20-30 percentage point reduction in readmission risk [18]. Another study of primary care and medical specialist follow-up visits found that amongst vulnerable patients, defined as either aged over 70 or with specified chronic health conditions, the period of largest contribution to readmission reduction was the first 10 days after discharge [16].

Our findings are consistent with this evidence and suggest that the benefits of timely follow-up also extend to follow-up provided by predominantly nurse-led CHS, such as community nursing or reablement teams. Whilst contact within seven days is also associated with significant reductions in hospital use, even faster follow-up within one day of discharge appears to be most beneficial for readmission reduction in this context. While previous studies have focused on specific patient groups, such as those with certain chronic health conditions, elderly patients or surgical discharges, our study is the first to quantify this effect using a nationally representative dataset, capturing multiple service types delivered to a broad population of patients discharged from hospital.

### Implications for policy

This study’s findings support the longstanding policy strategy of strengthening community-based care provision as a means of reducing the burden on hospital services. Earlier CHS contact was associated with lower odds of ED attendance and readmission, aligning with existing NHS guidance which emphasises the role of timely community follow-up to support safe discharge and recovery [31]. Our stratified analyses by primary diagnoses suggest that the strength of associations vary across clinical groups, which may help inform future service planning.

Although our study period predates the COVID-19 pandemic, which accelerated the adoption of remote consultations and monitoring of patients in the community through services such as virtual wards [46, 47], the underlying care model remains similar. Post-discharge care delivered through CHS remains the standard pathway for patients requiring short-term support after hospital discharge. The recent release of the 10 Year Health Plan for England (2025) has placed renewed emphasis on shifting care out of hospitals and into the community [35]. Our findings regarding the benefits of timely CHS contact and hospital use are therefore particularly relevant as the NHS seeks to expand its CHS provision.

However, realising these benefits will likely require targeted investment in CHS, which has historically experienced slower funding growth [48] and persistent workforce shortages [49], potentially constraining the delivery of timely and effective post-discharge care. Recent policy commitments to increase staffing and resources to CHS may help address these structural challenges [35]. Translating patient-level reductions in hospital activity into system-level impacts may also require complementary changes within the hospital sector [33]. Without adjustments to supply of services or payment incentives for hospitals, current activity-based payment systems within the NHS will continue to incentivise hospitals to fill any capacity which is released by post-discharge CHS care.

Policymakers could also consider approaches to support timely post-discharge CHS follow-up, such as the existing 72-hour follow-up requirement for discharges from inpatient mental health services [50]. Alternatively, financial incentives could be used to encourage timely care, as seen in several other health systems [51, 52]. For example, the use of Medicare transitional care management codes, which provided enhanced reimbursement for post-discharge clinician visits within seven or 14 days, were found to be associated with lower costs and mortality in the United States [52]. While our findings are derived from the English NHS, they may nevertheless be relevant to other healthcare systems where similar nurse-led community services support patients following hospital discharge.

Further research using quasi-experimental approaches could provide causal evidence on the impact of timely CHS contact on subsequent hospital use. Future research might also explore which types or intensities of CHS follow-up care are most effective in reducing subsequent hospital risk, the extent to which CHS care prevents versus merely delays subsequent hospital use, and the impact on wider outcomes such as patient satisfaction. As CHS data continues to improve, additional research is also needed to understand variation in service delivery and outcomes across different providers, including more specialised and independent sector providers.

### Strengths and limitations

This is the first study to assess the relationship between the timing of care contacts with CHS and hospital use outcomes at the patient-level. Using data across multiple providers covering a broad range of community services enabled analysis of a large, representative patient cohort and real-world CHS delivery across England. Our analyses adjusted for a comprehensive set of patient risk-adjusters from the index admission records, including clinical diagnoses, comorbidities, and socioeconomic deprivation. The findings were robust to landmark analyses restricting outcomes to days 8-30 post-discharge and alternative comparison groups. We used regression adjustment to control for observable confounders rather than propensity score methods as methodological studies suggest multiple logistic regression is preferable in terms of performance with large samples [53] and gives more precise estimates [54].

Despite comprehensive adjustment for patient case-mix and clinical complexity, unmeasured confounding remains possible. CHS contact is not randomly assigned and patients at higher risk may have been prioritised for earlier contact, introducing confounding by indication and selection bias. Descriptive statistics support this; patients seen within one day of discharge are older and have more comorbidities than those with no contact within 30 days. The strengthening of the estimated associations after covariate adjustment for contact within one day of discharge, particularly when patient demographic and diagnostic characteristics are introduced, suggests our estimates likely represent a conservative estimate of the true effect if residual confounding due to patient severity remains. Patients receiving a contact between two and seven days appear more similar to those with no contact, and estimated associations were more stable before and after adjustment, suggesting that unmeasured confounding may be less likely to bias the estimated effect for this group.

Social, community and cultural factors, such as informal carer support, access to social care, community resources and norms influencing healthcare engagement, may influence both the likelihood of receiving and attending post-discharge CHS contact and subsequent hospital use, but are not captured in administrative data. However, patient engagement is unlikely to be a major source of residual bias, as contacts not occurring due to patient cancellations, no shows, or late arrivals were infrequent (3.4%). Instead, limited CHS provider capacity likely explains why many referrals did not receive contact within 30 days of discharge, given the well documented workforce shortages facing the sector [49].We retained the first eligible admission-referral episode per patient, excluding subsequent episodes which occurred in 9% of patients. Effects may differ for subsequent admission-referral episodes so our estimates may not generalise to the effect of timely CHS contact following subsequent repeat admissions within a short timeframe. We did not distinguish between related and unrelated emergency readmissions, as diagnosis-based definitions would risk misclassifying clinically related events, particularly in this heterogeneous population. However, as unrelated admissions are expected to occur at similar rates across groups, the comparator design accounts for the underlying rate. Although we adjusted for patient case mix using 19 higher-level diagnostic ICD-10 clusters, some heterogeneity may remain within these groups.

Finally, the CSDS is a relatively new dataset with variation in completeness across Trusts. We restricted to Trusts with relatively high data quality, but variation in data recording likely remains.

## Conclusions

Timely post-discharge care from CHS, particularly within one day of discharge, is associated with substantially lower odds of ED attendance and hospital readmission. These findings highlight how strengthening CHS provision and ensuring rapid follow-up after discharge may support improved patient outcomes and help reduce pressure on hospital services.

## Supplementary information

Supplementary Information: Additional file 1: Figure S1. Sample selection. Table S1–Table S8

## Data Availability

The Community Services Dataset and Hospital Episode Statistics data used in this study were obtained from NHS England, restrictions apply to the availability of these data, which were used under license for the current study and therefore are not publicly available. The authors are not permitted to share this data. All other data used in this study is publicly available and referenced within the manuscript and supplementary material.

## Abbreviations

ACSC: Ambulatory care sensitive condition
CHS: Community health services
CSDS: Community services dataset
ED: Emergency department
HES: Hospital episode statistics
IMD: Indices of multiple deprivation
LSOA: Lower layer super output area
