# Supplementary material for: The impact of timely community health services contact following hospital discharge on subsequent hospital use in England: retrospective observational study
Source: BMC Med. 2026 Jul 15;24:557. doi: 10.1186/s12916-026-05015-3 (PMC13651089; doi:10.1186/s12916-026-05015-3)
Supplement: Supplementary file 1 [file 12916_2026_5015_MOESM1_ESM.docx]

**The impact of timely community health services contact following hospital discharge on subsequent hospital use: Retrospective observational study**

**Additional file 1**

Figure S1. Sample selection

CHS referrals with referral source of ‘acute inpatient or outpatient unit’

N=221,435 referrals (N=150,365 unique individuals)

Excluded, referral not linked to a relevant single index admission (N=125,904)

- No matching admission record (n=49,258)

- Referral date before index admission date or referral date greater than 30 days after discharge date (n=38,017)

- Referral matched to same index admission (n=38,629)

Matching referrals to a relevant index admission

Unique referral-admission episodes

N=96,531 referrals (N=84,813 unique individuals)

First referral per individual

Excluded multiple subsequent referrals for the same individual (N=11,717)

# Analysis sample

N=80,138 individuals

N=84,813 individuals referred to CHS following discharge from inpatient hospital admission

Excluded (N=4675)

- Outcome on same day as discharge n=4,649
- Missing data n=26

Data cleaning

Analysis

Main analysis sample

N=63,019 individuals

Excluded from main analysis (N=17,119) with contact between 8-30 days.

Included in supplementary analyses.

**Table S1. Descriptive statistics including individuals contacted between eight and 30 days post-discharge**

|  | Contact within 1 day (N=32,866) | Contact between 2 and 7 days (N=12,579) | Contact between 8 and 30 days (N=17,119) | No contact within 30 days (N=17,574) | Total (N=80,138) |
| --- | --- | --- | --- | --- | --- |
| ED attendance within 30 days of discharge | 6298 (19.2%) | 2364 (18.8%) | 5537 (32.3%) | 4191 (23.8%) | 12853 (20.4%) |
| Emergency re-admission within 30 days of discharge | 5853 (17.8%) | 2272 (18.1%) | 5919 (34.6%) | 3984 (22.7%) | 12109 (19.2%) |
| Re-admission for ACSC within 30 days of discharge | 1597 (4.9%) | 591 (4.7%) | 1471 (8.6%) | 1014 (5.8%) | 3202 (5.1%) |
| Age (Mean (SD)) | 75.0 (15.1) | 70.0 (16.7) | 68.2 (17.0) | 69.6 (17.9) | 72.5 (16.4) |
| Age group |  |  |  |  |  |
| Aged 18-30 | 519 (1.6%) | 409 (3.3%) | 647 (3.8%) | 794 (4.5%) | 1722 (2.7%) |
| Aged 30-39 | 658 (2.0%) | 452 (3.6%) | 762 (4.5%) | 813 (4.6%) | 1923 (3.1%) |
| Aged 40-49 | 1162 (3.5%) | 653 (5.2%) | 1034 (6.0%) | 957 (5.4%) | 2772 (4.4%) |
| Aged 50-59 | 2604 (7.9%) | 1418 (11.3%) | 2187 (12.8%) | 1788 (10.2%) | 5810 (9.2%) |
| Aged 60-69 | 4478 (13.6%) | 2287 (18.2%) | 3200 (18.7%) | 2802 (15.9%) | 9567 (15.2%) |
| Aged 70-79 | 8567 (26.1%) | 3388 (26.9%) | 4602 (26.9%) | 4554 (25.9%) | 16509 (26.2%) |
| Aged 80-89 | 10730 (32.6%) | 3036 (24.1%) | 3647 (21.3%) | 4412 (25.1%) | 18178 (28.8%) |
| Aged 90-99 | 4067 (12.4%) | 910 (7.2%) | 1025 (6.0%) | 1431 (8.1%) | 6408 (10.2%) |
| Aged 100+ | 81 (0.2%) | 26 (0.2%) | 15 (0.1%) | 23 (0.1%) | 130 (0.2%) |
| Sex |  |  |  |  |  |
| Male | 15282 (46.5%) | 6494 (51.6%) | 8667 (50.6%) | 8489 (48.3%) | 30265 (48.0%) |
| Female | 17584 (53.5%) | 6085 (48.4%) | 8452 (49.4%) | 9085 (51.7%) | 32754 (52.0%) |
| Major ethnic group |  |  |  |  |  |
| Unknown | 671 (2.0%) | 421 (3.3%) | 595 (3.5%) | 499 (2.8%) | 1591 (2.5%) |
| White | 28800 (87.6%) | 10691 (85.0%) | 14348 (83.8%) | 15004 (85.4%) | 54495 (86.5%) |
| Mixed | 473 (1.4%) | 197 (1.6%) | 322 (1.9%) | 415 (2.4%) | 1085 (1.7%) |
| Asian | 295 (0.9%) | 128 (1.0%) | 232 (1.4%) | 261 (1.5%) | 684 (1.1%) |
| Black | 2627 (8.0%) | 1142 (9.1%) | 1622 (9.5%) | 1395 (7.9%) | 5164 (8.2%) |
| Primary diagnosis of index admission |  |  |  |  |  |
| Blood disorders | 222 (0.7%) | 79 (0.6%) | 297 (1.7%) | 204 (1.2%) | 505 (0.8%) |
| Cancer | 2707 (8.2%) | 1121 (8.9%) | 2753 (16.1%) | 1858 (10.6%) | 5686 (9.0%) |
| Digestive system | 1892 (5.8%) | 564 (4.5%) | 969 (5.7%) | 994 (5.7%) | 3450 (5.5%) |
| Endocrine, nutritional and metabolic disease | 1052 (3.2%) | 282 (2.2%) | 307 (1.8%) | 477 (2.7%) | 1811 (2.9%) |
| Heart | 2528 (7.7%) | 2046 (16.3%) | 2038 (11.9%) | 1956 (11.1%) | 6530 (10.4%) |
| Infectious disease | 1082 (3.3%) | 256 (2.0%) | 309 (1.8%) | 408 (2.3%) | 1746 (2.8%) |
| Injuries / fractures | 5175 (15.7%) | 1726 (13.7%) | 1959 (11.4%) | 2371 (13.5%) | 9272 (14.7%) |
| Liver | 436 (1.3%) | 139 (1.1%) | 198 (1.2%) | 216 (1.2%) | 791 (1.3%) |
| Mental illness | 659 (2.0%) | 96 (0.8%) | 179 (1.0%) | 262 (1.5%) | 1017 (1.6%) |
| Musculoskeletal disorders | 3454 (10.5%) | 2071 (16.5%) | 2829 (16.5%) | 2095 (11.9%) | 7620 (12.1%) |
| Nervous system | 767 (2.3%) | 235 (1.9%) | 356 (2.1%) | 447 (2.5%) | 1449 (2.3%) |
| Other | 1718 (5.2%) | 502 (4.0%) | 825 (4.8%) | 780 (4.4%) | 3000 (4.8%) |
| Perinatal | 24 (0.1%) | 17 (0.1%) | 16 (0.1%) | 16 (0.1%) | 57 (0.1%) |
| Pregnancy related | 118 (0.4%) | 116 (0.9%) | 361 (2.1%) | 536 (3.0%) | 770 (1.2%) |
| Renal / genitourinary | 2555 (7.8%) | 781 (6.2%) | 891 (5.2%) | 1285 (7.3%) | 4621 (7.3%) |
| Respiratory | 3837 (11.7%) | 1074 (8.5%) | 1380 (8.1%) | 1736 (9.9%) | 6647 (10.5%) |
| Sense organs | 335 (1.0%) | 147 (1.2%) | 301 (1.8%) | 267 (1.5%) | 749 (1.2%) |
| Skin | 1295 (3.9%) | 333 (2.6%) | 324 (1.9%) | 430 (2.4%) | 2058 (3.3%) |
| Vascular / circulatory | 3010 (9.2%) | 994 (7.9%) | 827 (4.8%) | 1236 (7.0%) | 5240 (8.3%) |
| Count of Elixhauser comorbidities (Mean (SD)) | 2.5 (1.7) | 2.2 (1.7) | 2.0 (1.7) | 2.1 (1.7) | 2.3 (1.7) |
| Presence of Elixhauser comorbidities |  |  |  |  |  |
| Congestive heart failure | 4064 (12.4%) | 1271 (10.1%) | 1448 (8.5%) | 1648 (9.4%) | 6983 (11.1%) |
| Cardiac arrhythmia | 8708 (26.5%) | 2728 (21.7%) | 3104 (18.1%) | 3766 (21.4%) | 15202 (24.1%) |
| Valvular disease | 2625 (8.0%) | 1055 (8.4%) | 1022 (6.0%) | 1132 (6.4%) | 4812 (7.6%) |
| Pulmonary circulation disorders | 488 (1.5%) | 164 (1.3%) | 179 (1.0%) | 182 (1.0%) | 834 (1.3%) |
| Peripheral vascular disease | 1665 (5.1%) | 552 (4.4%) | 592 (3.5%) | 694 (3.9%) | 2911 (4.6%) |
| Hypertension uncomplicated | 16311 (49.6%) | 5824 (46.3%) | 7210 (42.1%) | 7884 (44.9%) | 30019 (47.6%) |
| Hypertension complicated | 44 (0.1%) | 17 (0.1%) | 22 (0.1%) | 30 (0.2%) | 91 (0.1%) |
| Paralysis | 1085 (3.3%) | 293 (2.3%) | 244 (1.4%) | 441 (2.5%) | 1819 (2.9%) |
| Other neurologic disorders | 2817 (8.6%) | 812 (6.5%) | 966 (5.6%) | 1269 (7.2%) | 4898 (7.8%) |
| Chronic pulmonary disease | 7095 (21.6%) | 2479 (19.7%) | 3351 (19.6%) | 3451 (19.6%) | 13025 (20.7%) |
| Diabetes uncomplicated | 6420 (19.5%) | 2271 (18.1%) | 2804 (16.4%) | 3114 (17.7%) | 11805 (18.7%) |
| Diabetes complicated | 1062 (3.2%) | 294 (2.3%) | 387 (2.3%) | 523 (3.0%) | 1879 (3.0%) |
| Hypothyroidism | 3055 (9.3%) | 972 (7.7%) | 1226 (7.2%) | 1356 (7.7%) | 5383 (8.5%) |
| Renal failure | 5100 (15.5%) | 1558 (12.4%) | 1917 (11.2%) | 2252 (12.8%) | 8910 (14.1%) |
| Liver disease | 890 (2.7%) | 282 (2.2%) | 389 (2.3%) | 478 (2.7%) | 1650 (2.6%) |
| Peptic ulcer disease excluding bleeding | 144 (0.4%) | 45 (0.4%) | 58 (0.3%) | 45 (0.3%) | 234 (0.4%) |
| Lymphoma | 203 (0.6%) | 69 (0.5%) | 119 (0.7%) | 104 (0.6%) | 376 (0.6%) |
| Metastatic cancer | 1521 (4.6%) | 491 (3.9%) | 1292 (7.5%) | 900 (5.1%) | 2912 (4.6%) |
| Solid tumor without metastasis | 1875 (5.7%) | 573 (4.6%) | 1002 (5.9%) | 845 (4.8%) | 3293 (5.2%) |
| Rheumatoid arthritis/collagen | 1817 (5.5%) | 657 (5.2%) | 763 (4.5%) | 882 (5.0%) | 3356 (5.3%) |
| Coagulopathy | 357 (1.1%) | 89 (0.7%) | 142 (0.8%) | 160 (0.9%) | 606 (1.0%) |
| Obesity | 2719 (8.3%) | 1365 (10.9%) | 1818 (10.6%) | 1605 (9.1%) | 5689 (9.0%) |
| Weight loss | 574 (1.7%) | 132 (1.0%) | 174 (1.0%) | 255 (1.5%) | 961 (1.5%) |
| Fluid and electrolyte disorders | 4521 (13.8%) | 1059 (8.4%) | 1177 (6.9%) | 1679 (9.6%) | 7259 (11.5%) |
| Blood loss anemia | 35 (0.1%) | 10 (0.1%) | 16 (0.1%) | 14 (0.1%) | 59 (0.1%) |
| Deficiency anemia | 1457 (4.4%) | 387 (3.1%) | 469 (2.7%) | 541 (3.1%) | 2385 (3.8%) |
| Alcohol abuse | 1331 (4.0%) | 428 (3.4%) | 513 (3.0%) | 609 (3.5%) | 2368 (3.8%) |
| Drug abuse | 169 (0.5%) | 65 (0.5%) | 94 (0.5%) | 121 (0.7%) | 355 (0.6%) |
| Psychoses | 273 (0.8%) | 75 (0.6%) | 95 (0.6%) | 135 (0.8%) | 483 (0.8%) |
| Depression | 3034 (9.2%) | 1070 (8.5%) | 1366 (8.0%) | 1528 (8.7%) | 5632 (8.9%) |
| Index of Multiple Deprivation (IMD) Decile |  |  |  |  |  |
| 1 (most deprived) | 1751 (5.3%) | 664 (5.3%) | 876 (5.1%) | 907 (5.2%) | 3322 (5.3%) |
| 2 | 2532 (7.7%) | 939 (7.5%) | 1320 (7.7%) | 1333 (7.6%) | 4804 (7.6%) |
| 3 | 3001 (9.1%) | 1099 (8.7%) | 1593 (9.3%) | 1772 (10.1%) | 5872 (9.3%) |
| 4 | 3358 (10.2%) | 1340 (10.7%) | 1886 (11.0%) | 1955 (11.1%) | 6653 (10.6%) |
| 5 | 3927 (11.9%) | 1528 (12.1%) | 2147 (12.5%) | 2397 (13.6%) | 7852 (12.5%) |
| 6 | 4274 (13.0%) | 1577 (12.5%) | 2032 (11.9%) | 2289 (13.0%) | 8140 (12.9%) |
| 7 | 3370 (10.3%) | 1331 (10.6%) | 1888 (11.0%) | 1957 (11.1%) | 6658 (10.6%) |
| 8 | 3534 (10.8%) | 1379 (11.0%) | 1843 (10.8%) | 1786 (10.2%) | 6699 (10.6%) |
| 9 | 3613 (11.0%) | 1368 (10.9%) | 1833 (10.7%) | 1821 (10.4%) | 6802 (10.8%) |
| 10 (least deprived) | 3506 (10.7%) | 1354 (10.8%) | 1701 (9.9%) | 1357 (7.7%) | 6217 (9.9%) |
| Index admission mode was emergency | 26519 (80.7%) | 8176 (65.0%) | 8858 (51.7%) | 11520 (65.6%) | 46215 (73.3%) |
| Discharged to usual place of residence | 28973 (88.2%) | 11771 (93.6%) | 15994 (93.4%) | 15664 (89.1%) | 56408 (89.5%) |
| Month of discharge |  |  |  |  |  |
| Apr-19 | 4635 (14.1%) | 1590 (12.6%) | 2434 (14.2%) | 1703 (9.7%) | 7928 (12.6%) |
| May-19 | 4720 (14.4%) | 1584 (12.6%) | 2228 (13.0%) | 1781 (10.1%) | 8085 (12.8%) |
| Jun-19 | 4109 (12.5%) | 1372 (10.9%) | 2060 (12.0%) | 1637 (9.3%) | 7118 (11.3%) |
| Jul-19 | 3930 (12.0%) | 1428 (11.4%) | 2105 (12.3%) | 2226 (12.7%) | 7584 (12.0%) |
| Aug-19 | 3512 (10.7%) | 1384 (11.0%) | 1819 (10.6%) | 2042 (11.6%) | 6938 (11.0%) |
| Sep-19 | 2963 (9.0%) | 1384 (11.0%) | 1990 (11.6%) | 2020 (11.5%) | 6367 (10.1%) |
| Oct-19 | 3222 (9.8%) | 1341 (10.7%) | 1989 (11.6%) | 2153 (12.3%) | 6716 (10.7%) |
| Nov-19 | 3031 (9.2%) | 1356 (10.8%) | 1475 (8.6%) | 2333 (13.3%) | 6720 (10.7%) |
| Dec-19 | 2744 (8.3%) | 1140 (9.1%) | 1019 (6.0%) | 1679 (9.6%) | 5563 (8.8%) |
| Day of week of discharge |  |  |  |  |  |
| Sunday | 1646 (5.0%) | 660 (5.2%) | 1067 (6.2%) | 940 (5.3%) | 3246 (5.2%) |
| Monday | 4844 (14.7%) | 1626 (12.9%) | 2689 (15.7%) | 2655 (15.1%) | 9125 (14.5%) |
| Tuesday | 6127 (18.6%) | 1957 (15.6%) | 3060 (17.9%) | 3151 (17.9%) | 11235 (17.8%) |
| Wednesday | 6238 (19.0%) | 1942 (15.4%) | 3065 (17.9%) | 3037 (17.3%) | 11217 (17.8%) |
| Thursday | 6164 (18.8%) | 1829 (14.5%) | 2908 (17.0%) | 3024 (17.2%) | 11017 (17.5%) |
| Friday | 5603 (17.0%) | 2958 (23.5%) | 2870 (16.8%) | 3225 (18.4%) | 11786 (18.7%) |
| Saturday | 2244 (6.8%) | 1607 (12.8%) | 1460 (8.5%) | 1542 (8.8%) | 5393 (8.6%) |

**Table S2. Logistic regressions of time to community health services contact on hospital use, fully adjusted models with all regression coefficients presented (Model 5 in Table 2)**

|  | ED attendance within 30 days of discharge | Re-admission within 30 days of discharge | ACSC readmission within 30 days of discharge |
| --- | --- | --- | --- |
|  | OR [95% CI] | OR [95% CI] | OR [95% CI] |
|  |  |  |  |
| Ref: No contact within follow-up period | - | - | - |
|  | - | - | - |
|  |  |  |  |
| Contact within 1 day | 0.674^***^ | 0.615^***^ | 0.688^***^ |
|  | [0.640,0.709] | [0.583,0.649] | [0.627,0.755] |
|  |  |  |  |
| Contact between 2 and 7 days | 0.804^***^ | 0.775^***^ | 0.818^***^ |
|  | [0.756,0.855] | [0.728,0.825] | [0.730,0.916] |
|  |  |  |  |
| Aged 18-30 | 0.690^**^ | 0.672^**^ | 0.654 |
|  | [0.551,0.864] | [0.530,0.853] | [0.400,1.069] |
|  |  |  |  |
| Aged 30-39 | 0.692^***^ | 0.647^***^ | 0.584^*^ |
|  | [0.562,0.853] | [0.518,0.808] | [0.362,0.942] |
|  |  |  |  |
| Aged 40-49 | 0.627^***^ | 0.603^***^ | 0.482^***^ |
|  | [0.532,0.738] | [0.507,0.717] | [0.330,0.704] |
|  |  |  |  |
| Aged 50-59 | 0.695^***^ | 0.726^***^ | 0.661^***^ |
|  | [0.621,0.778] | [0.647,0.816] | [0.525,0.832] |
|  |  |  |  |
| Aged 60-69 | 0.728^***^ | 0.807^***^ | 0.948 |
|  | [0.664,0.797] | [0.736,0.885] | [0.805,1.116] |
|  |  |  |  |
| Aged 70-79 | 0.904^**^ | 0.918^*^ | 0.940 |
|  | [0.839,0.975] | [0.850,0.992] | [0.820,1.078] |
|  |  |  |  |
| Aged 80-89 | - | - | - |
|  | - | - | - |
|  |  |  |  |
| Aged 90-99 | 1.075 | 1.036 | 1.114 |
|  | [0.961,1.202] | [0.922,1.163] | [0.914,1.358] |
|  |  |  |  |
| Aged 100+ | 1.330 | 0.946 | 3.312^*^ |
|  | [0.537,3.295] | [0.349,2.564] | [1.010,10.86] |
|  |  |  |  |
| Sex: male | - | - | - |
|  | - | - | - |
|  |  |  |  |
| Sex: female | 0.931 | 0.952 | 1.118 |
|  | [0.866,1.001] | [0.883,1.026] | [0.983,1.272] |
|  |  |  |  |
| Aged 18-30 # Male | - | - | - |
|  | - | - | - |
|  |  |  |  |
| Aged 18-30 # Female | 1.476^**^ | 1.206 | 1.264 |
|  | [1.105,1.971] | [0.883,1.646] | [0.676,2.363] |
|  |  |  |  |
| Aged 30-39 # Male | - | - | - |
|  | - | - | - |
|  |  |  |  |
| Aged 30-39 # Female | 1.228 | 1.218 | 1.678 |
|  | [0.936,1.611] | [0.913,1.626] | [0.936,3.009] |
|  |  |  |  |
| Aged 40-49 # Male | - | - | - |
|  | - | - | - |
|  |  |  |  |
| Aged 40-49 # Female | 1.307^*^ | 1.370^**^ | 1.495 |
|  | [1.050,1.626] | [1.089,1.722] | [0.919,2.432] |
|  |  |  |  |
| Aged 50-59 # Male | - | - | - |
|  | - | - | - |
|  |  |  |  |
| Aged 50-59 # Female | 1.085 | 1.031 | 1.180 |
|  | [0.926,1.270] | [0.877,1.212] | [0.865,1.609] |
|  |  |  |  |
| Aged 60-69 # Male | - | - | - |
|  | - | - | - |
|  |  |  |  |
| Aged 60-69 # Female | 1.127 | 1.101 | 0.925 |
|  | [0.991,1.282] | [0.966,1.254] | [0.733,1.166] |
|  |  |  |  |
| Aged 70-79 # Male | - | - | - |
|  | - | - | - |
|  |  |  |  |
| Aged 70-79 # Female | 1.004 | 1.033 | 1.047 |
|  | [0.904,1.115] | [0.927,1.150] | [0.868,1.264] |
|  |  |  |  |
| Aged 80-89 # Male | - | - | - |
|  | - | - | - |
|  |  |  |  |
| Aged 80-89 # Female | - | - | - |
|  | - | - | - |
|  |  |  |  |
| Aged 90-99 # Male | - | - | - |
|  | - | - | - |
|  |  |  |  |
| Aged 90-99 # Female | 0.974 | 1.032 | 0.916 |
|  | [0.845,1.123] | [0.891,1.196] | [0.712,1.178] |
|  |  |  |  |
| Aged 100+ # Male | - | - | - |
|  | - | - | - |
|  |  |  |  |
| Aged 100+ # Female | 0.930 | 0.992 | 0.336 |
|  | [0.337,2.570] | [0.326,3.021] | [0.0809,1.397] |
|  |  |  |  |
| Ethnicity: Unknown | 0.662^***^ | 0.694^***^ | 0.567^**^ |
|  | [0.568,0.772] | [0.593,0.811] | [0.399,0.805] |
|  |  |  |  |
| Ethnicity: White | - | - | - |
|  |  |  |  |
|  |  |  |  |
| Ethnicity: Mixed | 1.008 | 0.956 | 1.152 |
|  | [0.861,1.181] | [0.809,1.129] | [0.862,1.540] |
|  |  |  |  |
| Ethnicity: Asian | 1.110 | 0.977 | 0.787 |
|  | [0.920,1.339] | [0.798,1.196] | [0.517,1.197] |
|  |  |  |  |
| Ethnicity: Black | 0.824^***^ | 0.874^**^ | 0.790^**^ |
|  | [0.758,0.895] | [0.802,0.951] | [0.668,0.935] |
|  |  |  |  |
| Primary diagnosis: Blood disorders | 1.575^***^ | 1.972^***^ | 1.510^*^ |
|  | [1.278,1.940] | [1.597,2.436] | [1.001,2.278] |
|  |  |  |  |
| Primary diagnosis: Cancer | 1.615^***^ | 2.325^***^ | 1.643^***^ |
|  | [1.460,1.786] | [2.098,2.576] | [1.333,2.025] |
|  |  |  |  |
| Primary diagnosis: | 1.513^***^ | 1.937^***^ | 1.501^***^ |
|  | [1.370,1.670] | [1.750,2.144] | [1.225,1.839] |
|  |  |  |  |
| Primary diagnosis: Endocrine, nutritional and metabolic disease | 1.333^***^ | 1.676^***^ | 2.874^***^ |
|  | [1.176,1.512] | [1.476,1.903] | [2.341,3.528] |
|  |  |  |  |
| Primary diagnosis: Heart | 1.151^**^ | 1.183^***^ | 2.099^***^ |
|  | [1.053,1.258] | [1.077,1.299] | [1.780,2.476] |
|  |  |  |  |
| Primary diagnosis: Infectious disease | 1.326^***^ | 1.663^***^ | 1.886^***^ |
|  | [1.169,1.503] | [1.464,1.888] | [1.503,2.366] |
|  |  |  |  |
| Primary diagnosis: Injuries / fractures | - | - | - |
|  |  |  |  |
|  |  |  |  |
| Primary diagnosis: Liver | 1.749^***^ | 2.476^***^ | 1.238 |
|  | [1.469,2.082] | [2.081,2.945] | [0.836,1.832] |
|  |  |  |  |
| Primary diagnosis: Mental illness | 1.487^***^ | 1.662^***^ | 1.943^***^ |
|  | [1.274,1.736] | [1.417,1.951] | [1.463,2.581] |
|  |  |  |  |
| Primary diagnosis: Musculoskeletal disorders | 1.051 | 1.094 | 1.041 |
|  | [0.962,1.149] | [0.994,1.204] | [0.854,1.270] |
|  |  |  |  |
| Primary diagnosis: Nervous system | 1.215^**^ | 1.275^**^ | 1.345 |
|  | [1.050,1.406] | [1.091,1.489] | [0.991,1.825] |
|  |  |  |  |
| Primary diagnosis: Other | 1.396^***^ | 1.761^***^ | 1.251 |
|  | [1.249,1.560] | [1.572,1.973] | [0.976,1.604] |
|  |  |  |  |
| Primary diagnosis: Perinatal | 2.109^*^ | 1.091 | 0.961 |
|  | [1.097,4.055] | [0.457,2.603] | [0.128,7.235] |
|  |  |  |  |
| Primary diagnosis: Pregnancy related | 0.615^***^ | 0.593^**^ | 0.166^**^ |
|  | [0.469,0.807] | [0.432,0.814] | [0.0514,0.535] |
|  |  |  |  |
| Primary diagnosis: Renal / genitourinary | 1.604^***^ | 1.779^***^ | 2.116^***^ |
|  | [1.466,1.755] | [1.619,1.955] | [1.778,2.519] |
|  |  |  |  |
| Primary diagnosis: Respiratory | 1.420^***^ | 1.638^***^ | 3.024^***^ |
|  | [1.309,1.540] | [1.504,1.783] | [2.609,3.506] |
|  |  |  |  |
| Primary diagnosis: Sense organs | 1.596^***^ | 1.510^***^ | 1.483^*^ |
|  | [1.331,1.914] | [1.241,1.836] | [1.014,2.170] |
|  |  |  |  |
| Primary diagnosis: Skin | 1.102 | 1.631^***^ | 3.114^***^ |
|  | [0.972,1.251] | [1.441,1.847] | [2.562,3.785] |
|  |  |  |  |
| Primary diagnosis: Vascular / circulatory | 1.023 | 1.071 | 1.033 |
|  | [0.930,1.125] | [0.968,1.184] | [0.843,1.267] |
|  |  |  |  |
| congestive heart failure | 1.169^***^ | 1.149^***^ | 1.302^***^ |
|  | [1.097,1.247] | [1.075,1.227] | [1.174,1.443] |
|  |  |  |  |
| cardiac arrhythmia | 1.182^***^ | 1.150^***^ | 1.205^***^ |
|  | [1.126,1.241] | [1.094,1.210] | [1.107,1.312] |
|  |  |  |  |
| valvular disease | 1.054 | 1.068 | 0.982 |
|  | [0.977,1.138] | [0.988,1.155] | [0.864,1.117] |
|  |  |  |  |
| pulmonary circulation disorders | 1.128 | 1.238^*^ | 1.237 |
|  | [0.958,1.327] | [1.052,1.456] | [0.970,1.578] |
|  |  |  |  |
| peripheral vascular disease | 1.101^*^ | 1.134^**^ | 1.307^***^ |
|  | [1.005,1.207] | [1.033,1.245] | [1.131,1.510] |
|  |  |  |  |
| hypertension uncomplicated | 1.012 | 0.982 | 0.959 |
|  | [0.970,1.057] | [0.940,1.026] | [0.888,1.036] |
|  |  |  |  |
| hypertension complicated | 0.778 | 0.702 | 0.516 |
|  | [0.468,1.295] | [0.418,1.181] | [0.185,1.439] |
|  |  |  |  |
| paralysis | 1.033 | 1.017 | 1.011 |
|  | [0.914,1.168] | [0.894,1.156] | [0.798,1.282] |
|  |  |  |  |
| other neurologic disorders | 1.215^***^ | 1.141^***^ | 1.076 |
|  | [1.131,1.307] | [1.058,1.231] | [0.942,1.229] |
|  |  |  |  |
| chronic pulmonary disease | 1.175^***^ | 1.140^***^ | 1.369^***^ |
|  | [1.120,1.233] | [1.085,1.198] | [1.261,1.487] |
|  |  |  |  |
| diabetes uncomplicated | 1.118^***^ | 1.137^***^ | 1.196^***^ |
|  | [1.062,1.177] | [1.079,1.198] | [1.094,1.308] |
|  |  |  |  |
| diabetes complicated | 1.262^***^ | 1.348^***^ | 1.565^***^ |
|  | [1.128,1.411] | [1.203,1.509] | [1.317,1.859] |
|  |  |  |  |
| hypothyroidism | 1.059 | 1.049 | 1.045 |
|  | [0.987,1.136] | [0.975,1.127] | [0.923,1.182] |
|  |  |  |  |
| renal failure | 1.203^***^ | 1.270^***^ | 1.319^***^ |
|  | [1.135,1.274] | [1.198,1.347] | [1.201,1.450] |
|  |  |  |  |
| liver disease | 1.208^**^ | 1.262^***^ | 1.040 |
|  | [1.071,1.363] | [1.117,1.425] | [0.832,1.299] |
|  |  |  |  |
| peptic ulcer disease excluding bleeding | 1.302 | 1.122 | 1.095 |
|  | [0.967,1.752] | [0.824,1.529] | [0.638,1.879] |
|  |  |  |  |
| lymphoma | 1.149 | 1.389^**^ | 0.920 |
|  | [0.903,1.461] | [1.096,1.759] | [0.593,1.427] |
|  |  |  |  |
| metastatic cancer | 1.202^***^ | 1.396^***^ | 1.026 |
|  | [1.084,1.333] | [1.263,1.543] | [0.836,1.260] |
|  |  |  |  |
| solid tumor without metastatis | 1.207^***^ | 1.301^***^ | 1.110 |
|  | [1.105,1.318] | [1.191,1.420] | [0.944,1.306] |
|  |  |  |  |
| rheumatoid arthritis/collagen | 1.057 | 1.117^*^ | 1.058 |
|  | [0.969,1.153] | [1.023,1.220] | [0.912,1.228] |
|  |  |  |  |
| coagulopathy | 1.091 | 1.174 | 1.005 |
|  | [0.899,1.325] | [0.964,1.428] | [0.696,1.453] |
|  |  |  |  |
| obesity | 0.916^*^ | 0.897^**^ | 0.805^**^ |
|  | [0.849,0.989] | [0.829,0.970] | [0.695,0.932] |
|  |  |  |  |
| weight loss | 1.050 | 1.064 | 1.014 |
|  | [0.900,1.226] | [0.908,1.246] | [0.770,1.336] |
|  |  |  |  |
| fluid and electrolyte disorders | 1.069^*^ | 1.082^*^ | 1.058 |
|  | [1.005,1.137] | [1.016,1.153] | [0.953,1.175] |
|  |  |  |  |
| blood loss anemia | 1.232 | 1.314 | 2.344^*^ |
|  | [0.695,2.184] | [0.739,2.338] | [1.024,5.364] |
|  |  |  |  |
| deficiency anemia | 1.033 | 1.056 | 1.038 |
|  | [0.934,1.143] | [0.952,1.171] | [0.871,1.237] |
|  |  |  |  |
| alcohol abuse | 1.124^*^ | 1.041 | 0.920 |
|  | [1.010,1.251] | [0.931,1.164] | [0.747,1.134] |
|  |  |  |  |
| drug abuse | 1.617^***^ | 1.275 | 1.110 |
|  | [1.261,2.074] | [0.970,1.674] | [0.670,1.837] |
|  |  |  |  |
| psychoses | 1.363^**^ | 1.235 | 1.422^*^ |
|  | [1.103,1.684] | [0.989,1.541] | [1.002,2.019] |
|  |  |  |  |
| depression | 1.235^***^ | 1.119^**^ | 1.188^**^ |
|  | [1.154,1.322] | [1.043,1.202] | [1.054,1.340] |
|  |  |  |  |
| IMD decile 1 (Most deprived) | - | - | - |
|  |  |  |  |
|  |  |  |  |
| IMD decile 2 | 0.953 | 0.990 | 0.991 |
|  | [0.855,1.062] | [0.885,1.107] | [0.820,1.196] |
|  |  |  |  |
| IMD decile 3 | 0.909 | 0.989 | 0.895 |
|  | [0.818,1.009] | [0.888,1.101] | [0.744,1.076] |
|  |  |  |  |
| IMD decile 4 | 0.857^**^ | 0.901 | 0.811^*^ |
|  | [0.773,0.951] | [0.809,1.003] | [0.674,0.975] |
|  |  |  |  |
| IMD decile 5 | 0.836^***^ | 0.900 | 0.867 |
|  | [0.755,0.926] | [0.811,1.000] | [0.725,1.037] |
|  |  |  |  |
| IMD decile 6 | 0.821^***^ | 0.877^*^ | 0.777^**^ |
|  | [0.741,0.908] | [0.789,0.974] | [0.648,0.931] |
|  |  |  |  |
| IMD decile 7 | 0.867^**^ | 0.931 | 0.874 |
|  | [0.780,0.962] | [0.836,1.037] | [0.726,1.052] |
|  |  |  |  |
| IMD decile 8 | 0.820^***^ | 0.895^*^ | 0.862 |
|  | [0.738,0.911] | [0.803,0.997] | [0.715,1.038] |
|  |  |  |  |
| IMD decile 9 | 0.789^***^ | 0.850^**^ | 0.821^*^ |
|  | [0.710,0.877] | [0.762,0.948] | [0.679,0.993] |
|  |  |  |  |
| IMD decile 10 (Least deprived) | 0.716^***^ | 0.796^***^ | 0.736^**^ |
|  | [0.641,0.799] | [0.711,0.892] | [0.604,0.898] |
|  |  |  |  |
| Index admission emergency flag | 1.419^***^ | 1.568^***^ | 1.822^***^ |
|  | [1.334,1.510] | [1.470,1.672] | [1.600,2.074] |
|  |  |  |  |
| April 2019 | - | - | - |
|  |  |  |  |
|  |  |  |  |
| May | 0.918^*^ | 0.874^***^ | 0.867^*^ |
|  | [0.849,0.991] | [0.808,0.946] | [0.756,0.995] |
|  |  |  |  |
| June | 0.904^*^ | 0.886^**^ | 0.898 |
|  | [0.834,0.979] | [0.817,0.961] | [0.779,1.035] |
|  |  |  |  |
| July | 0.872^***^ | 0.850^***^ | 0.811^**^ |
|  | [0.805,0.943] | [0.784,0.921] | [0.703,0.936] |
|  |  |  |  |
| August | 0.909^*^ | 0.813^***^ | 0.813^**^ |
|  | [0.839,0.985] | [0.748,0.883] | [0.701,0.942] |
|  |  |  |  |
| September | 0.855^***^ | 0.813^***^ | 0.828^*^ |
|  | [0.787,0.930] | [0.747,0.886] | [0.712,0.963] |
|  |  |  |  |
| October | 0.882^**^ | 0.784^***^ | 0.796^**^ |
|  | [0.813,0.958] | [0.720,0.853] | [0.686,0.924] |
|  |  |  |  |
| November | 0.889^**^ | 0.829^***^ | 0.885 |
|  | [0.819,0.965] | [0.762,0.901] | [0.765,1.024] |
|  |  |  |  |
| December | 0.795^***^ | 0.724^***^ | 0.720^***^ |
|  | [0.727,0.868] | [0.661,0.793] | [0.613,0.847] |
|  |  |  |  |
| Sunday | - | - | - |
|  |  |  |  |
|  |  |  |  |
| Monday | 1.040 | 1.026 | 1.029 |
|  | [0.938,1.152] | [0.922,1.142] | [0.846,1.253] |
|  |  |  |  |
| Tuesday | 1.004 | 1.019 | 1.046 |
|  | [0.907,1.110] | [0.918,1.132] | [0.864,1.267] |
|  |  |  |  |
| Wednesday | 1.069 | 1.086 | 1.154 |
|  | [0.967,1.182] | [0.978,1.205] | [0.954,1.395] |
|  |  |  |  |
| Thursday | 1.014 | 1.060 | 1.113 |
|  | [0.917,1.122] | [0.954,1.177] | [0.920,1.347] |
|  |  |  |  |
| Friday | 0.969 | 0.999 | 1.034 |
|  | [0.877,1.072] | [0.900,1.109] | [0.854,1.251] |
|  |  |  |  |
| Saturday | 0.986 | 1.007 | 1.005 |
|  | [0.882,1.104] | [0.896,1.131] | [0.812,1.244] |
|  |  |  |  |
| Discharged to usual place of residence | 1.259^***^ | 1.408^***^ | 1.382^***^ |
|  | [1.175,1.349] | [1.308,1.515] | [1.211,1.577] |
|  |  |  |  |
| Observations | 63019 | 63019 | 63019 |
| C-Statistic | 0.637 | 0.654 | 0.716 |

Individuals with no contact during the follow-up period are the comparator group. Coefficients are odds ratios. 95% confidence intervals in brackets. C-statistic (area under the receiver operating characteristic curve) measures the model’s discriminatory power. ED = Emergency Department, ACSC = Ambulatory care sensitive conditions. ^*^ *p* < 0.05, ^**^ *p* < 0.01, ^***^ *p* < 0.001

**Table S3. Fully adjusted logistic regressions of time to community health services contact on hospital use, stratified by age group and index admission type**

|  |  | Full sample | Under 65 | 65 and over | Elective admission | Emergency admission |
| --- | --- | --- | --- | --- | --- | --- |
|  |  | OR [95% CI] | OR [95% CI] | OR [95% CI] | OR [95% CI] | OR [95% CI] |
| ED attendance within 30 days | Ref: No contact within follow-up period | - | - | - | - | - |
|  |  |  |  |  |  |  |
|  | Contact within 1 day of discharge | 0.674^***^ | 0.688^***^ | 0.661^***^ | 0.629^***^ | 0.682^***^ |
|  |  | [0.640,0.709] | [0.614,0.770] | [0.623,0.701] | [0.561,0.706] | [0.643,0.723] |
|  |  |  |  |  |  |  |
|  | Contact between 2 and 7 days | 0.804^***^ | 0.898 | 0.777^***^ | 0.817^**^ | 0.817^***^ |
|  |  | [0.756,0.855] | [0.792,1.020] | [0.724,0.834] | [0.724,0.922] | [0.760,0.879] |
|  |  |  |  |  |  |  |
|  | Observations | 63,019 | 16010 | 47009 | 16804 | 46215 |
|  | C-statistic | 0.637 | 0.677 | 0.627 | 0.673 | 0.625 |
| Emergency readmission within 30 days | Ref: No contact within follow-up period | - | - | - | - | - |
|  |  |  |  |  |  |  |
|  | Contact within 1 day of discharge | 0.615^***^ | 0.616^***^ | 0.605^***^ | 0.530^***^ | 0.634^***^ |
|  |  | [0.583,0.649] | [0.547,0.693] | [0.570,0.642] | [0.470,0.597] | [0.596,0.673] |
|  |  |  |  |  |  |  |
|  | Contact between 2 and 7 days | 0.775^***^ | 0.846^*^ | 0.761^***^ | 0.752^***^ | 0.802^***^ |
|  |  | [0.728,0.825] | [0.741,0.965] | [0.708,0.818] | [0.663,0.854] | [0.745,0.864] |
|  |  |  |  |  |  |  |
|  | Observations | 63,019 | 16010 | 47009 | 16804 | 46215 |
|  | C-statistic | 0.654 | 0.711 | 0.639 | 0.707 | 0.638 |
| Emergency ACSC admission within 30 days | Ref: No contact within follow-up period | - | - | - | - | - |
|  |  |  |  |  |  |  |
|  | Contact within 1 day of discharge | 0.688^***^ | 0.688^***^ | 0.680^***^ | 0.609^***^ | 0.699^***^ |
|  |  | [0.627,0.755] | [0.551,0.860] | [0.614,0.754] | [0.470,0.789] | [0.632,0.773] |
|  |  |  |  |  |  |  |
|  | Contact between 2 and 7 days | 0.818^***^ | 0.945 | 0.786^***^ | 0.872 | 0.828^**^ |
|  |  | [0.730,0.916] | [0.727,1.228] | [0.692,0.893] | [0.659,1.155] | [0.731,0.939] |
|  |  |  |  |  |  |  |
|  | Observations | 63,019 | 16010 | 47009 | 16804 | 46215 |
|  | C-statistic | 0.716 | 0.803 | 0.698 | 0.756 | 0.696 |

Individuals with no contact during the follow-up period are the comparator group. Models are adjusted for patient demographics covariates: age group (ten-year age bands), sex, ethnic group and IMD decile, diagnosis covariates: 19 primary diagnosis clusters, 30 indicators for the presence of each Elixhauser comorbidity, indicator for index admission being an emergency, whether discharged to usual place of residence, indicators for calendar month and day of week of discharge, and indicators for CHS provider of referral and acute care provider of index admission. Coefficients are odds ratios. 95% confidence intervals in brackets. C-statistic (area under the receiver operating characteristic curve) measures the model’s discriminatory power. ED = Emergency Department, ACSC = Ambulatory care sensitive conditions. ^*^ *p* < 0.05, ^**^ *p* < 0.01, ^***^ *p* < 0.001

**Table S4. Fully adjusted logistic regressions of time to community health services contact on hospital use, stratified by primary diagnosis group**

|  |  | Full sample | Cancer | Digestive system | Heart | Injuries | Musculoskeletal | Renal | Respiratory | Vascular |
| --- | --- | --- | --- | --- | --- | --- | --- | --- | --- | --- |
|  |  | OR [95% CI] | OR [95% CI] | OR [95% CI] | OR [95% CI] | OR [95% CI] | OR [95% CI] | OR [95% CI] | OR [95% CI] | OR [95% CI] |
| ED attendance within 30 days | Ref: No contact within follow-up period | - | - | - | - | - | - | - | - | - |
|  |  |  |  |  |  |  |  |  |  |  |
|  | Contact within 1 day of discharge | 0.674^***^ | 0.595^***^ | 0.667^***^ | 0.759^***^ | 0.789^***^ | 0.696^***^ | 0.608^***^ | 0.667^***^ | 0.717^***^ |
|  |  | [0.640,0.709] | [0.511,0.694] | [0.551,0.808] | [0.649,0.887] | [0.690,0.902] | [0.595,0.813] | [0.517,0.714] | [0.584,0.761] | [0.601,0.854] |
|  |  |  |  |  |  |  |  |  |  |  |
|  | Contact between 2 and 7 days | 0.804^***^ | 0.836 | 1.001 | 0.773^**^ | 0.813^*^ | 0.649^***^ | 0.814^*^ | 0.819^*^ | 0.958 |
|  |  | [0.756,0.855] | [0.697,1.003] | [0.786,1.276] | [0.654,0.913] | [0.681,0.970] | [0.539,0.781] | [0.662,0.999] | [0.687,0.975] | [0.773,1.188] |
|  |  |  |  |  |  |  |  |  |  |  |
|  | Observations | 63,019 | 5686 | 3450 | 6530 | 9272 | 7620 | 4621 | 6647 | 5240 |
|  | C-statistic | 0.637 | 0.610 | 0.631 | 0.638 | 0.654 | 0.681 | 0.633 | 0.600 | 0.641 |
| Emergency readmission within 30 days | Ref: No contact within follow-up period | - | - | - | - | - | - | - | - | - |
|  |  |  |  |  |  |  |  |  |  |  |
|  | Contact within 1 day of discharge | 0.615^***^ | 0.533^***^ | 0.645^***^ | 0.684^***^ | 0.770^***^ | 0.692^***^ | 0.554^***^ | 0.650^***^ | 0.587^***^ |
|  |  | [0.583,0.649] | [0.461,0.617] | [0.534,0.778] | [0.580,0.807] | [0.668,0.889] | [0.584,0.821] | [0.470,0.653] | [0.568,0.743] | [0.488,0.705] |
|  |  |  |  |  |  |  |  |  |  |  |
|  | Contact between 2 and 7 days | 0.775^***^ | 0.827^*^ | 1.025 | 0.708^***^ | 0.794^*^ | 0.717^**^ | 0.763^*^ | 0.855 | 0.937 |
|  |  | [0.728,0.825] | [0.696,0.983] | [0.808,1.300] | [0.594,0.845] | [0.654,0.963] | [0.585,0.880] | [0.618,0.942] | [0.717,1.019] | [0.749,1.172] |
|  |  |  |  |  |  |  |  |  |  |  |
|  | Observations | 63,019 | 5686 | 3450 | 6530 | 9272 | 7620 | 4621 | 6647 | 5240 |
|  | C-statistic | 0.654 | 0.632 | 0.629 | 0.674 | 0.661 | 0.721 | 0.648 | 0.609 | 0.666 |
| Emergency ACSC admission within 30 days | Ref: No contact within follow-up period | - | - | - | - | - | - | - | - | - |
|  |  |  |  |  |  |  |  |  |  |  |
|  | Contact within 1 day of discharge | 0.688^***^ | 0.628^**^ | 0.527^**^ | 0.582^***^ | 0.690^**^ | 0.810 | 0.665^**^ | 0.754^**^ | 0.684^*^ |
|  |  | [0.627,0.755] | [0.443,0.890] | [0.358,0.777] | [0.453,0.748] | [0.524,0.909] | [0.563,1.165] | [0.504,0.877] | [0.625,0.909] | [0.470,0.995] |
|  |  |  |  |  |  |  |  |  |  |  |
|  | Contact between 2 and 7 days | 0.818^***^ | 1.030 | 1.088 | 0.742^*^ | 0.641^*^ | 0.868 | 0.697 | 0.996 | 0.733 |
|  |  | [0.730,0.916] | [0.691,1.535] | [0.677,1.746] | [0.566,0.973] | [0.424,0.970] | [0.555,1.359] | [0.478,1.016] | [0.781,1.270] | [0.451,1.190] |
|  |  |  |  |  |  |  |  |  |  |  |
|  | Observations | 63,019 | 5686 | 3450 | 6530 | 9272 | 7620 | 4621 | 6647 | 5240 |
|  | C-statistic | 0.716 | 0.706 | 0.754 | 0.730 | 0.743 | 0.793 | 0.709 | 0.660 | 0.752 |

Individuals with no contact during the follow-up period are the comparator group. Models are adjusted for patient demographics covariates: age group (ten-year age bands), sex, ethnic group and IMD decile, diagnosis covariates: 19 primary diagnosis clusters, 30 indicators for the presence of each Elixhauser comorbidity, indicator for index admission being an emergency, whether discharged to usual place of residence, indicators for calendar month and day of week of discharge, and indicators for CHS provider of referral and acute care provider of index admission. Coefficients are odds ratios. 95% confidence intervals in brackets. C-statistic (area under the receiver operating characteristic curve) measures the model’s discriminatory power. ED = Emergency Department, ACSC = Ambulatory care sensitive conditions. ^*^ *p* < 0.05, ^**^ *p* < 0.01, ^***^ *p* < 0.001

**Table S5. Logistic regressions of time to CHS contact on hospital use 8 to 30 days following discharge**

|  |  | Unadjusted | Adjusted | Landmark analysis^†^:  Unadjusted | Landmark analysis^†^: Adjusted |
| --- | --- | --- | --- | --- | --- |
|  |  | OR [95% CI] | OR [95% CI] | OR [95% CI] | OR [95% CI] |
| Emergency department attendance between 8-30 days of discharge | Ref: No contact within follow-up period | - | - | - | - |
|  |  |  |  |  |  |
|  | Contact within 1 day of discharge | 0.780^***^ | 0.701^***^ | 0.758^***^ | 0.669^***^ |
|  |  | [0.739,0.824] | [0.657,0.747] | [0.718,0.801] | [0.627,0.714] |
|  |  |  |  |  |  |
|  | Contact between 2 and 7 days | 0.638^***^ | 0.688^***^ | 0.630^***^ | 0.687^***^ |
|  |  | [0.593,0.687] | [0.636,0.745] | [0.585,0.679] | [0.634,0.745] |
|  |  |  |  |  |  |
|  | Observations | 63019 | 63019 | 57465 | 57465 |
|  | C-statistic | 0.541 | 0.634 | 0.543 | 0.645 |
| Emergency readmission between 8-30 days of discharge | Ref: No contact within follow-up period | - | - | - | - |
|  |  |  |  |  |  |
|  | Contact within 1 day of discharge | 0.758^***^ | 0.655^***^ | 0.737^***^ | 0.620^***^ |
|  |  | [0.717,0.801] | [0.614,0.699] | [0.697,0.780] | [0.581,0.663] |
|  |  |  |  |  |  |
|  | Contact between 2 and 7 days | 0.590^***^ | 0.604^***^ | 0.589^***^ | 0.606^***^ |
|  |  | [0.546,0.636] | [0.556,0.655] | [0.546,0.636] | [0.558,0.659] |
|  |  |  |  |  |  |
|  | Observations | 63019 | 63019 | 57883 | 57883 |
|  | C-statistic | 0.548 | 0.653 | 0.548 | 0.664 |
| Emergency ACSC admission between 8-30 days of discharge | Ref: No contact within follow-up period | - | - | - | - |
|  |  |  |  |  |  |
|  | Contact within 1 day of discharge | 0.812^***^ | 0.686^***^ | 0.810^***^ | 0.678^***^ |
|  |  | [0.735,0.897] | [0.611,0.769] | [0.734,0.895] | [0.604,0.761] |
|  |  |  |  |  |  |
|  | Contact between 2 and 7 days | 0.635^***^ | 0.633^***^ | 0.637^***^ | 0.635^***^ |
|  |  | [0.553,0.728] | [0.546,0.733] | [0.555,0.730] | [0.548,0.737] |
|  |  |  |  |  |  |
|  | Observations | 63019 | 63019 | 61803 | 61803 |
|  | C-statistic | 0.540 | 0.717 | 0.540 | 0.720 |
|  | Other covariates included in regressions: |  |  |  |  |
|  | Demographics | No | Yes | No | Yes |
|  | Diagnoses | No | Yes | No | Yes |
|  | Month and day of discharge | No | Yes | No | Yes |
|  | Provider indicators | No | Yes | No | Yes |

^†^ Individuals with an outcome event within 7 days from discharge (the landmark time) are excluded. Individuals with no contact during the follow-up period are the comparator group. Patient demographics covariates: age group (ten-year age bands), sex, ethnic group and IMD decile. 19 primary diagnosis clusters, 30 indicators for the presence of each Elixhauser comorbidity, indicator for index admission being an emergency, whether discharged to usual place of residence. Month and day discharge covariates: Indicators for calendar month and day of week of discharge. Provider indicators: Indicators for CHS provider of referral and acute care provider of index admission. Coefficients are odds ratios. 95% confidence intervals in brackets. C-statistic (area under the receiver operating characteristic curve) measures the model’s discriminatory power. ED = Emergency Department, ACSC = Ambulatory care sensitive conditions. ^*^ *p* < 0.05, ^**^ *p* < 0.01, ^***^ *p* < 0.001

**Table S6. Logistic regressions of time to CHS contact on hospital use within 30 and within 90 days of discharge amongst patients referred before 31^st^ October 2019.**

|  |  | 30-days unadjusted | 30-days adjusted | 90-days unadjusted | 90-days adjusted |
| --- | --- | --- | --- | --- | --- |
|  |  | OR [95% CI] | OR [95% CI] | OR [95% CI] | OR [95% CI] |
| Emergency department attendance within 30 days | Ref: No contact within follow-up period | - | - | - | - |
|  |  |  |  |  |  |
|  | Contact within 1 day of discharge | 0.745^***^ | 0.672^***^ | 0.852^***^ | 0.751^***^ |
|  |  | [0.709,0.783] | [0.634,0.712] | [0.816,0.890] | [0.714,0.791] |
|  |  |  |  |  |  |
|  | Contact between 2 and 7 days | 0.739^***^ | 0.817^***^ | 0.741^***^ | 0.820^***^ |
|  |  | [0.693,0.788] | [0.762,0.875] | [0.701,0.784] | [0.771,0.872] |
|  |  |  |  |  |  |
|  | C-statistic | 0.530 | 0.640 | 0.527 | 0.647 |
| Emergency readmission within 30 days | Ref: No contact within follow-up period | - | - | - | - |
|  |  |  |  |  |  |
|  | Contact within 1 day of discharge | 0.737^***^ | 0.618^***^ | 0.876^***^ | 0.717^***^ |
|  |  | [0.700,0.776] | [0.582,0.656] | [0.838,0.916] | [0.680,0.755] |
|  |  |  |  |  |  |
|  | Contact between 2 and 7 days | 0.754^***^ | 0.788^***^ | 0.780^***^ | 0.817^***^ |
|  |  | [0.707,0.805] | [0.734,0.846] | [0.737,0.826] | [0.768,0.870] |
|  |  |  |  |  |  |
|  | C-statistic | 0.531 | 0.656 | 0.522 | 0.663 |
| Preventable admission within 30 days | Ref: No contact within follow-up period | - | - | - | - |
|  |  |  |  |  |  |
|  | Contact within 1 day of discharge | 0.839^***^ | 0.696^***^ | 0.949 | 0.788^***^ |
|  |  | [0.766,0.920] | [0.627,0.773] | [0.885,1.018] | [0.726,0.854] |
|  |  |  |  |  |  |
|  | Contact between 2 and 7 days | 0.793^***^ | 0.821^**^ | 0.815^***^ | 0.836^***^ |
|  |  | [0.704,0.892] | [0.721,0.934] | [0.744,0.893] | [0.756,0.923] |
|  |  |  |  |  |  |
|  | C-statistic | 0.522 | 0.717 | 0.517 | 0.713 |
|  | Other covariates included in regressions: |  |  |  |  |
|  | Demographics | No | Yes | No | Yes |
|  | Month and day of discharge | No | Yes | No | Yes |
|  | Timing variables | No | Yes | No | Yes |
|  | Provider indicators | No | Yes | No | Yes |

N=50,191, referrals between 1^st^ April 2019 and 31^st^ October 2019. Individuals with no contact during the follow-up period are the comparator group. Patient demographics covariates: age group (ten-year age bands), sex, ethnic group and IMD decile. Diagnosis covariates 19 primary diagnosis clusters, 30 indicators for the presence of each Elixhauser comorbidity, indicator for index admission being an emergency, whether discharged to usual place of residence. Month and day discharge covariates: Indicators for calendar month and day of week of discharge. Provider indicators: Indicators for CHS provider of referral and acute care provider of index admission. Coefficients are odds ratios. 95% confidence intervals in brackets. C-statistic (area under the receiver operating characteristic curve) measures the model’s discriminatory power. ED = Emergency Department, ACSC = Ambulatory care sensitive conditions. ^*^ *p* < 0.05, ^**^ *p* < 0.01, ^***^ *p* < 0.001

**Table S7. Logistic regressions of time to CHS contact on hospital use, contact within one and two-to-seven days compared with no contact within seven days of discharge.**

|  |  | Unadjusted | Fully adjusted |
| --- | --- | --- | --- |
|  |  | OR [95% CI] | OR [95% CI] |
| ED attendance within 30 days | Ref: No contact within follow-up period | - | - |
|  |  |  |  |
|  | Contact within 1 day of discharge | 0.608^***^ | 0.469^***^ |
|  |  | [0.587,0.631] | [0.450,0.489] |
|  |  |  |  |
|  | Contact between 2 and 7 days | 0.594^***^ | 0.587^***^ |
|  |  | [0.565,0.625] | [0.556,0.620] |
|  |  |  |  |
|  | C-statistic | 0.563 | 0.665 |
| Emergency readmission within 30 days | Ref: No contact within follow-up period | - | - |
|  |  |  |  |
|  | Contact within 1 day of discharge | 0.542^***^ | 0.392^***^ |
|  |  | [0.523,0.563] | [0.376,0.409] |
|  |  |  |  |
|  | Contact between 2 and 7 days | 0.552^***^ | 0.518^***^ |
|  |  | [0.524,0.581] | [0.491,0.548] |
|  |  |  |  |
|  | C-statistic | 0.576 | 0.689 |
| Emergency ACSC admission within 30 days | Ref: No contact within follow-up period | - | - |
|  |  |  |  |
|  | Contact within 1 day of discharge | 0.662^***^ | 0.474^***^ |
|  |  | [0.620,0.706] | [0.440,0.509] |
|  |  |  |  |
|  | Contact between 2 and 7 days | 0.639^***^ | 0.582^***^ |
|  |  | [0.583,0.701] | [0.528,0.643] |
|  |  |  |  |
|  | C-statistic | 0.553 | 0.730 |
|  | Other covariates included in regressions: |  |  |
|  | Patient demographics | No | Yes |
|  | Diagnoses | No | Yes |
|  | Month and day of discharge | No | Yes |
|  | Provider indicators | No | Yes |

N=80,138. Individuals with no contact within 7 days of discharge are the comparator group. Patient demographics covariates: age group (ten-year age bands), sex, ethnic group and IMD decile. Diagnosis covariates: 19 primary diagnosis clusters, 30 indicators for the presence of each Elixhauser comorbidity, indicator for index admission being an emergency, whether discharged to usual place of residence. Month and day discharge covariates: Indicators for calendar month and day of week of discharge. Provider indicators: Indicators for CHS provider of referral and acute care provider of index admission. Coefficients are odds ratios. 95% confidence intervals in brackets. C-statistic (area under the receiver operating characteristic curve) measures the model’s discriminatory power. ED = Emergency Department, ACSC = Ambulatory care sensitive conditions. ^*^ *p* < 0.05, ^**^ *p* < 0.01, ^***^ *p* < 0.001

**Table S8. Logistic regressions of time to community health services contact on hospital use amongst referrals between 1^st^ May 2019 and 31^st^ December 2019.**

|  |  | Unadjusted | Fully adjusted |
| --- | --- | --- | --- |
|  |  | OR [95% CI] | OR [95% CI] |
| ED attendance within 30 days | Ref: No contact within follow-up period | - | - |
|  |  |  |  |
|  | Contact within 1 day of discharge | 0.756^***^ | 0.678^***^ |
|  |  | [0.721,0.792] | [0.642,0.717] |
|  |  |  |  |
|  | Contact between 2 and 7 days | 0.730^***^ | 0.805^***^ |
|  |  | [0.687,0.776] | [0.753,0.860] |
|  |  |  |  |
|  | C-statistic | 0.532 | 0.637 |
| Emergency readmission within 30 days | Ref: No contact within follow-up period | - | - |
|  |  |  |  |
|  | Contact within 1 day of discharge | 0.726^***^ | 0.613^***^ |
|  |  | [0.691,0.762] | [0.579,0.649] |
|  |  |  |  |
|  | Contact between 2 and 7 days | 0.743^***^ | 0.779^***^ |
|  |  | [0.699,0.791] | [0.728,0.833] |
|  |  |  |  |
|  | C-statistic | 0.535 | 0.654 |
| Emergency ACSC admission within 30 days | Ref: No contact within follow-up period | - | - |
|  |  |  |  |
|  | Contact within 1 day of discharge | 0.810^***^ | 0.680^***^ |
|  |  | [0.742,0.884] | [0.615,0.752] |
|  |  |  |  |
|  | Contact between 2 and 7 days | 0.817^***^ | 0.850^**^ |
|  |  | [0.731,0.913] | [0.753,0.960] |
|  |  |  |  |
|  | C-statistic | 0.523 | 0.716 |
|  | Other covariates included in regressions: |  |  |
|  | Patient demographics | No | Yes |
|  | Diagnoses | No | Yes |
|  | Month and day of discharge | No | Yes |
|  | Provider indicators | No | Yes |

N=55,091 referrals between 1^st^ May 2019 and 31^st^ December 2019. Individuals with no contact within 7 days of discharge are the comparator group. Patient demographics covariates: age group (ten-year age bands), sex, ethnic group and IMD decile. Diagnosis covariates: 19 primary diagnosis clusters, 30 indicators for the presence of each Elixhauser comorbidity, indicator for index admission being an emergency, whether discharged to usual place of residence. Month and day discharge covariates: Indicators for calendar month and day of week of discharge. Provider indicators: Indicators for CHS provider of referral and acute care provider of index admission. Coefficients are odds ratios. 95% confidence intervals in brackets. C-statistic (area under the receiver operating characteristic curve) measures the model’s discriminatory power. ED = Emergency Department, ACSC = Ambulatory care sensitive conditions. ^*^ *p* < 0.05, ^**^ *p* < 0.01, ^***^ *p* < 0.001
